# Porous nanographene formation on γ-alumina nanoparticles *via* transition-metal-free methane activation†

**DOI:** 10.1039/d1sc06578e

**Published:** 2022-02-22

**Authors:** Masanori Yamamoto, Qi Zhao, Shunsuke Goto, Yu Gu, Takaaki Toriyama, Tomokazu Yamamoto, Hirotomo Nishihara, Alex Aziz, Rachel Crespo-Otero, Devis Di Tommaso, Masazumi Tamura, Keiichi Tomishige, Takashi Kyotani, Kaoru Yamazaki

**Affiliations:** Institute of Multidisciplinary Research for Advanced Materials, Tohoku University 2-1-1 Katahira, Aoba Sendai 980-8577 Japan yamamoto@mol-chem.com; Department of Chemistry, Queen Mary University of London Mile End Road London E1 4NS UK d.ditommaso@qmul.ac.uk; Graduate School of Engineering, Tohoku University 6-6-07 Aramaki, Aoba Sendai 980-8579 Japan; The Ultramicroscopy Research Center, Kyushu University Motooka 744, Nishi Fukuoka 819-0395 Japan; Institute for Materials Research, Tohoku University 2-1-1 Katahira, Aoba Sendai 980-8577 Japan

## Abstract

γ-Al_2_O_3_ nanoparticles promote pyrolytic carbon deposition of CH_4_ at temperatures higher than 800 °C to give single-walled nanoporous graphene (NPG) materials without the need for transition metals as reaction centers. To accelerate the development of efficient reactions for NPG synthesis, we have investigated early-stage CH_4_ activation for NPG formation on γ-Al_2_O_3_ nanoparticles *via* reaction kinetics and surface analysis. The formation of NPG was promoted at oxygen vacancies on (100) surfaces of γ-Al_2_O_3_ nanoparticles following surface activation by CH_4_. The kinetic analysis was well corroborated by a computational study using density functional theory. Surface defects generated as a result of surface activation by CH_4_ make it kinetically feasible to obtain single-layered NPG, demonstrating the importance of precise control of oxygen vacancies for carbon growth.

## Abstract 2

Oxygen vacancies on the (100) surface of γ-Al_2_O_3_ nanoparticles catalyse CH_4_-CVD for single-layered nanoporous graphenes with no transition metal reaction centre. The rate-limiting step is the proton transfer (PT) in the activation of CH_4_ on them.

## Graphical abstract

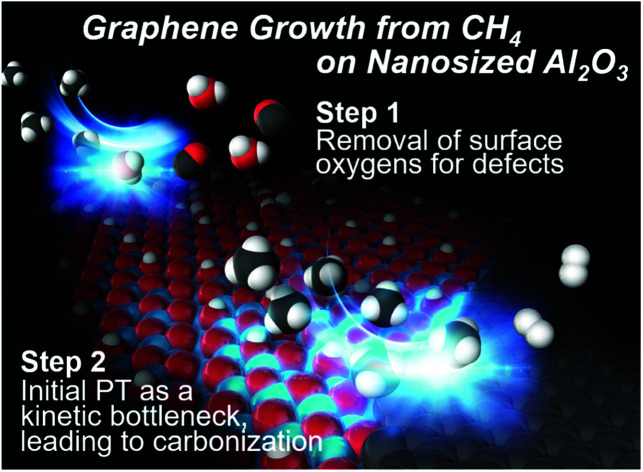

## Introduction

**Scheme 1:**
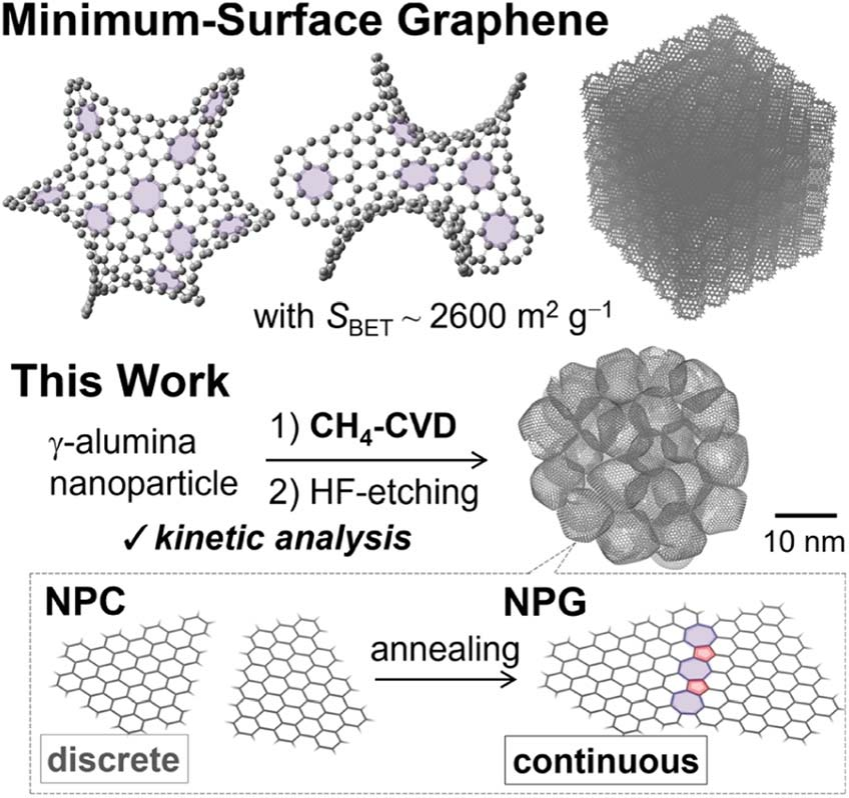
(top) Schematic of the minimum-surface graphene analogue originally reported by Mackay and Terrones.^6^ (bottom) Schematic of the synthesis of single-walled nanoporous carbon (NPC) and its conversion to single-walled nanoporous graphene (NPG) by the fusion of edge sites.^13–16^

Graphene is a two-dimensional (2D) allotrope of carbon arranged in a planar hexagonal lattice that displays high elasticity and electronic/thermal conductivity.^1^ When pentagons are introduced into the hexagonal frameworks, ring closure takes place with a positive curvature to give fullerenes.^2^ When 2D graphene is wrapped into a cylindrical structure, a 1D carbon nanotube could be obtained.^3–5^ In contrast, the synthesis of a three-dimensionally and periodically arranged single-walled graphene with a negative curvature has remained a challenge since Mackay and Terrones proposed an ideal 3D minimum-surface graphene structure (Scheme 1) in 1991,^6^ despite the recent advances in organic synthesis realizing small molecules with a few heptagons^7,8^ or octagons.^9,10^

In this regard, chemical vapor deposition (CVD) on templated materials^11–24^ is a prominent strategy for realizing 3D minimum-surface graphene. Especially, CVD on alumina nanoparticles (ANPs) that display high thermal stability^25^ as the templates gave nanoporous graphene (NPG) materials from CH_4._^14–17^ The NPGs have a 3D continuous and seamless nanostructure with a large surface area, approaching the ideal value of 2D graphene (2627 m^2^ g^−1^).^26^ NPGs also have fascinating features including high electrical conductivity,^16^ elastic and flexible nature,^15,17^ and unprecedentedly high electrochemical stability.^16,17^

The typical synthesis of NPG is performed as follows: a uniform carbon coating *via* CVD of CH_4_ at 900 °C on ANPs is followed by the removal of ANPs by chemical washing, and the subsequent high-temperature annealing at temperatures higher than 1600 °C under an inert atmosphere to give a single-walled NPG material (Scheme 1 and Fig. S1†).^14–17^ It is essential to use stable CH_4_ with a high bond dissociation energy (439 kJ mol^−1^)^27,28^ as a carbon source, rather than more reactive unsaturated hydrocarbons such as acetylene and propylene, for high-quality graphene formation by suppressing carbon stacking.

Understanding the type of reaction steps involved in the CH_4_-CVD process will represent a breakthrough in the synthesis of sophisticated nanoporous carbon materials, by using tastefully designed templates at lower temperatures *via* CH_4_-CVD. We recently reported the radical activation of CH_4_ on MgO,^24^ but the reaction mechanism on ANPs may vary owing to the differences in the geometry and electronic structure of the active site. Such a fundamental understanding of CH_4_ chemistry on the surfaces of metal oxides could also help us to efficiently activate hydrocarbons^29–49^ by controlling coke deposition at the molecular level.

In this work, we investigated the early-stage CH_4_ activation toward the formation of NPGs on γ-Al_2_O_3_ nanoparticles *via* reaction kinetics using thermogravimetry-mass spectrometry (TG-MS) and density functional theory (DFT), and surface analysis with high-resolution annular dark-field scanning transmission electron microscopy (ADF-STEM), temperature-programmed desorption (TPD) of H_2_O, and *in situ* infrared (IR) spectroscopy. We found that the formation of NPG is promoted at oxygen vacancies on (100) surfaces of γ-Al_2_O_3_, which are generated at temperatures higher than 800 °C in the presence of CH_4_. No transition metal reaction center was involved in CH_4_-CVD. This process is completely different from conventional methane activation catalysis for graphene^50–57^ and carbon nanotube^58–61^ growth at 1000 °C, oxidative^39–41^ and non-oxidative^42^ coupling to ethylene, and partial oxidation to methanol,^44–49^ which use transition metal elements as reaction centers. We first discuss the rate-limiting step of NPG formation on ANPs based on reaction kinetics using TG-MS and a reaction pathway search using DFT. We constructed a model (100) γ-Al_2_O_3_ surface based on high-resolution ADF-STEM, TPD of H_2_O, and *in situ* IR spectroscopy. Next, we discuss the strategy to further enhance the formation of NPG based on the surface activation process monitored by TG-MS and *in situ* IR spectroscopy. Details of the experimental and computational methods are provided in the ESI.†

## Results and discussion

### Kinetic analysis

**Fig. 1:**
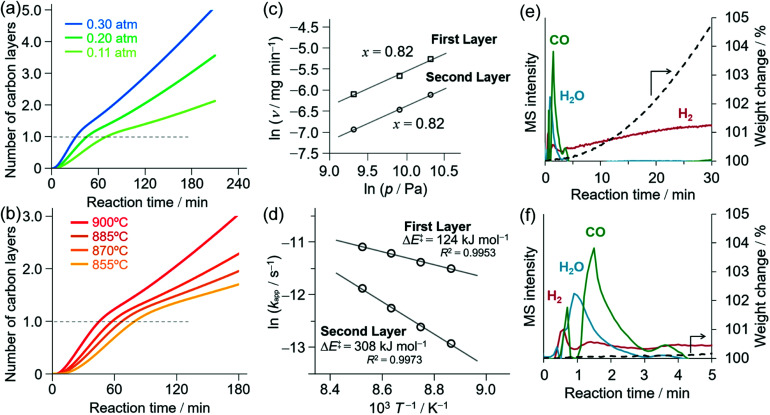
Kinetic analysis of CH_4_-CVD for porous nanographene. (a) Weight changes during CH_4_-CVD for various partial pressures of CH_4_ at 900 °C as monitored by TG. CH_4_ was introduced to the reactor at 0 min. (b) Weight changes during CH_4_-CVD on γ-ANPs at various temperatures as monitored by TG. (c) CH_4_ partial pressure dependence on the rate of carbon growth *v* at 900 °C. (d) Arrhenius plots for the first- and second-layer deposition. *P*/*P*_0_ = 0.2 for CH_4_ supply; the total rate of flow was fixed at 100 mL min^−1^. (e) TG-MS analysis of CH_4_-CVD under a steady flow of He (80 mL min^−1^) and CH_4_ (20 mL min^−1^) at 900 °C showing gas evolution for H_2_, H_2_O, and CO as well as the TG curve (dashed line). (f) Enlarged view of Fig. 1e showing the transient evolution of H_2_O and CO.

Fig. 1a–d shows the dependence of the rate of CH_4_-CVD on the partial pressure of CH_4_ and reaction temperature using TG. The vertical axis shows the nominal number of layers of deposited carbon on ANPs calculated by the specific surface area of ANPs.^17^ The rate of carbon growth was pseudo-first order with respect to the partial pressure of CH_4_ for both first- and second-layer depositions (Fig. 1c). The formation of single-layered nanographene at a specific time of reaction was confirmed by the red-shifted strong G′-band^17^ of Raman spectra (Fig. S1†). This pseudo-linear dependence of the rate of carbon growth on CH_4_ partial pressure suggests that the rate-limiting step of NPG growth may be the initial CH_4_ activation^52,62^ on the surface. The rate of carbon growth for the first layer was faster than those for the second and third layers, as shown in Fig. 1a and b. We evaluated the effective activation energies Δ*E*^≠^ for the first- and second-layer carbon growth from the Arrhenius plots as shown in Fig. 1d, and obtained Δ*E*^≠^ = 124 kJ mol^−1^ and 308 kJ mol^−1^, respectively. The latter value is in a good agreement with the activation energy for CH_4_ decomposition onto the surface of carbon films (303 kJ mol^−1^).^63^ Thus, the initial carbon deposition on the γ-ANP surface was kinetically more favorable than the subsequent carbon deposition on the deposited carbon surface, and this makes it feasible to selectively form single-walled porous nanographene. The carbon deposition proceeded after an induction time of 5–10 min. Transient evolutions of CO, H_2_, and H_2_O were also observed in the effluent gas of CH_4_-CVD reaction during the induction period as shown in Fig. 1e and f. To check the effect of H_2_ on the activation of oxide surfaces, we examined H_2_ treatment of ANPs at 900 °C for 30 min before CH_4_-CVD. However, there was no significant difference observed in the rate of reactions by TG (Fig. S2†). These indicate that CH_4_ chemically activated γ-ANPs and resulted in reactive surfaces for CH_4_-CVD.

### Surface analysis

To clarify the structure of the reaction center of the NPG growth on γ-ANP (first layer), we next investigated the structure of the reaction sites on γ-ANP by ADF-STEM, TPD of H_2_O, and *in situ* IR spectroscopy, and found that the oxygen vacancy sites of the partially hydrated (100) surface of γ-ANP are potential reaction centers.

**Fig. 2:**
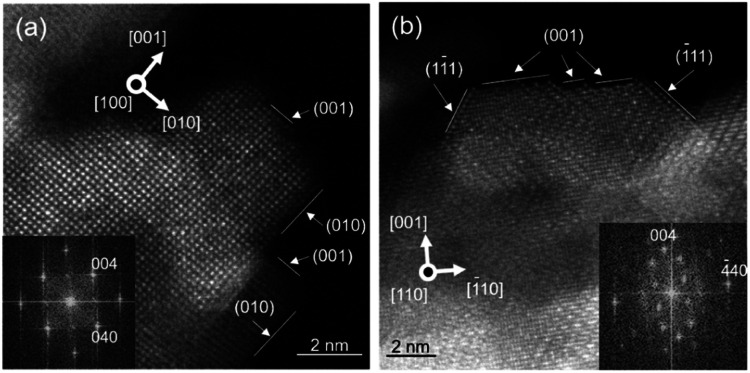
High resolution ADF-STEM image of γ-ANPs. (a) [100] and (b) [110] orientations. The insets of (a) and (b) are the corresponding FFT images.

The high-resolution ADF-STEM images (Fig. 2a, b and S3†) indicate that γ-ANPs had {100} as one of the main facets. The corresponding fast Fourier transformed (FFT) image was consistent with the neutron diffraction pattern for the (100) surface of γ-Al_2_O_3_.^64^ The γ-ANPs were prepared by hydrothermal synthesis, and the amount of transition metal impurities in the γ-ANPs was negligible according to our elemental analysis (for details, see the ESI†). We did not observe dispersion of any transition metal impurities on the surfaces in the ADF-STEM images.

**Fig. 3:**
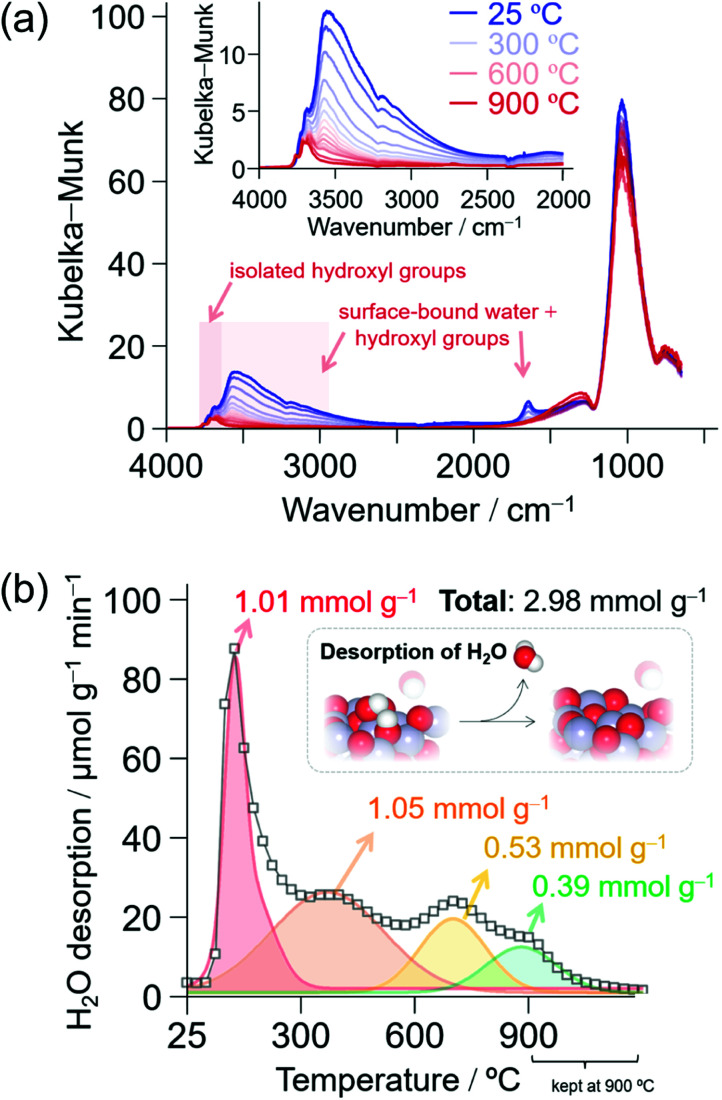
H_2_O desorption profile of γ-ANP: (a) Temperature dependence of IR spectra under a steady flow of Ar at 5 mL min^−1^. (b) TPD profile of H_2_O desorption from the surfaces of γ-ANP at 10 K min^−1^ quantified by GC (TCD). Gas: He flow at 200 mL min^−1^. The inset shows a schematic of water desorption from two protons and an oxide to give a surface defect. The thermal treatment under inert gas atmosphere was immediately followed by CH_4_-CVD (Fig. 1) by introducing CH_4_.

TG analysis of CH_4_-CVD suggests that reaction temperatures above 800 °C are essential for carbon growth from CH_4_. We analyzed the effects of temperature on the surface structure of γ-ANPs by IR and TPD (Fig. 3a and b) up to 900 °C. The IR spectra showed desorption of H_2_O and depletion of surface-bound hydroxyl groups (3800–3000 cm^−1^)^65,66^ as the operating temperature increased under a steady flow of inert gases. Water desorption became almost negligible after 30 min at 900 °C as shown in the TPD (Fig. 3b), but a sharp absorption band in the IR spectrum centered at 3701 cm^−1^ originating from “isolated” hydroxyl groups^25,67^ still remained (Fig. 3a). This indicates that terminal μ_1_-hydroxyl groups existed even at such high temperature. Although the isolated hydroxyl group was labile toward proton exchange upon exposure to CD_4_ at temperatures higher than 650 °C (Fig. S4 and S5†), no deposition of carbon occurred at lower temperatures. This supports that further activation of surfaces by the formation of oxygen vacancies,^68,69^ as observed by TG-MS (Fig. 1e and f) at higher temperatures in the presence of CH_4_, is crucial for carbon growth to take place.

### DFT calculations

To further examine the reaction mechanism, we constructed a partially dehydrated γ-Al_2_O_3_ (100) surface model with an oxygen vacancy. We evaluated the reaction pathway of the initial CH_4_ activation on the vacancy site using the plane-wave DFT and climbing-image nudged elastic band methods under periodic boundary conditions. We used the Perdew–Burke–Ernzerhof (PBE) exchange-correlation functional^70^ combined with Grimme's DFT-D3 empirical dispersion correction^71^ to account for the van der Waals interactions (PBE-D3). For details of the DFT calculations including the computational methods and structures of the intermediates and transition states involved in the CH_4_ activation on γ-Al_2_O_3_ (100), see Sections S1.4 and S3 of the ESI.†

We found that the dissociative adsorption of CH_4_ on the oxygen vacancy is the rate-limiting step, which is in agreement with our experiments, and that the dissociative addition undergoes in terms of the Lewis acid-base mechanism.^25,72–74^

We first examined CH_4_ activation on a 5-coordinated Al site (Fig. S6†).^66^ Methanol formation is expected on the oxide surface from CH_4_, and this is followed by desorption of the molecule to give an oxygen vacancy (4-coordinated Al site), as shown in Fig. S7.† The released methanol will decompose in the gas phase to afford CO and H_2_ at the operating temperatures of 800–900 °C,^75^ and this was confirmed by TG-MS analysis (Fig. 1f). With this in mind, we then investigated CH_4_ activation on the 4-coordinated Al site.

**Fig. 4:**
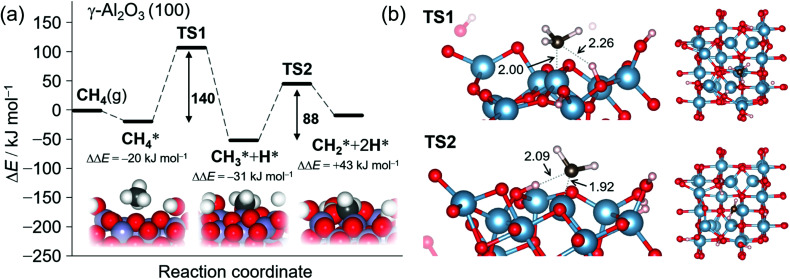
(a) Potential energy profile for the sequential C–H bond cleavage from a CH_4_ σ complex on a γ-Al_2_O_3_ (100) surface with a surface defect. The Al atom on the reaction site is set to be 4-coordinate. Activation energies were computed with the PBE-D3 functional and the corresponding structures of the initial, intermediate, and final states. The asterisk indicates the surface-bound species. Geometry for the oxygen defect is also shown in Fig. S7.† (b) Obtained structures of TS1 and TS2. Red: oxygen, steel blue; aluminum, black: carbon, and white: hydrogen atoms, with distances given in Ångströms.

Fig. 4a shows the calculated potential energy profile of CH_4_ activation on the partially dehydrated γ-Al_2_O_3_ (100) model surface (Fig. S7†), from the physisorption of CH_4_ to the formation of surface-bound methylene 
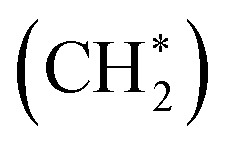
. The physisorption energy Δ*E*_ad_ is –20 kJ mol^−1^. The subsequent dissociative addition of adsorbed 
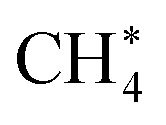
 gives a surface-bound methyl group 
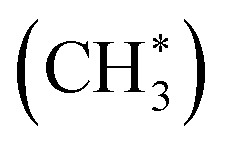
 and hydrogen atom (H*) with the activation energy Δ*E*_a,__TS1_ of 140 kJ mol^−1^. For surface reactions with small Δ*E*_ad_, as in the present case, the effective activation energy Δ*E*^≠^ of the overall reaction evaluated as the sum of Δ*E*_a,__TS1_ and Δ*E*_ad_ (ref. 76) is 120 kJ mol^−1^. This theoretical value is in excellent agreement with the experimental value of 124 kJ mol^−1^ (Fig. 1d).

**Fig. 5:**
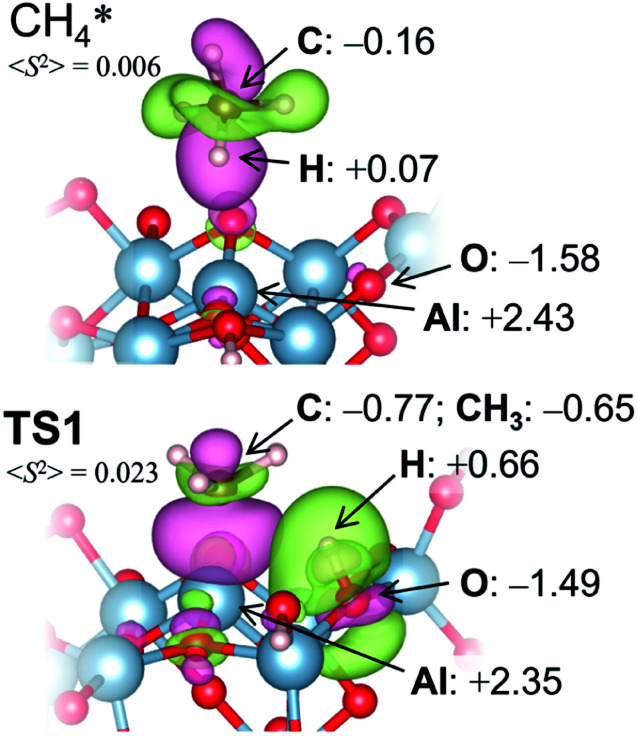
Charge difference profiles and Bader charges of selected atoms/groups on a γ-Al_2_O_3_ (100) surface with a defect. Green contour: positive charges, pink contour: negative charges.

Bader charges^77^ {*q*} of the transition state (TS) structure for the dissociative addition (TS1) in Fig. 5 clearly show that this reaction proceeds heterolytically following an acid-base mechanism, as suggested by the surface characterizations. H^*δ*+^ (*q* = +0.66) interacts with the oxygen Lewis base site (*q* = −1.49), while CH_3_^*δ*–^ (*q* = −0.65) is bound to the aluminum Lewis acid site (*q* = +2.35) at TS1. Pyramidal CH_3_^*δ*–^ at TS1 also supports the Lewis acid–base mechanism.^72–74^ The expected value for the square of the total spin angular momentum, <*S*^2^> = 0.023 at TS1, indicates significant electron pairing during the dissociative adsorption. The counter plot of the difference charge density in TS1 (Fig. 5) suggests that the large overlap between the doubly occupied 2p orbital at CH_4_ and the vacant 3p orbital at the bare Al atom promotes the reaction.

After dissociative adsorption, the surface 
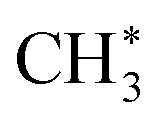
 is converted to 
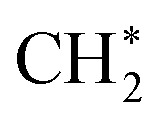
 with a considerably lower activation energy (Δ*E*_a_ = 88 kJ mol^−1^) *via* a proton transfer transition state TS2. The formed reactive 
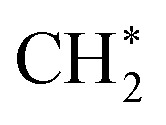
 species could then undergo a coupling reaction with another 
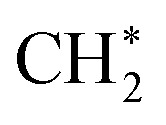
, which would result in the formation of 
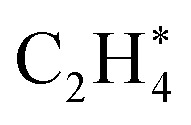
 and longer hydrocarbons on the surface, as reported in the conversion of surface bound-methylene to a class of graphene materials on Al_2_O_3_ surfaces.^78^ Although we cannot exclude the possibility that 
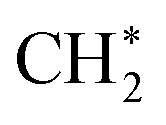
 will desorb to the gas phase,^42,79^ selective formation of single-layered NPG (Fig. S1†) indicates that this surface reaction rather than a gas-phase reaction enhances the first-layer deposition of carbon.

The activation energy for the first step of CH_4_ activation 
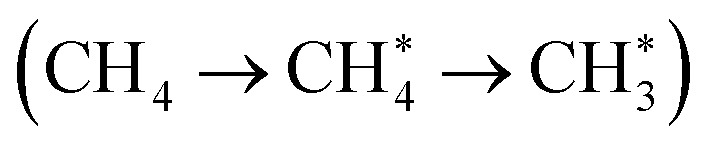
 on a non-defective hydrated γ-Al_2_O_3_ (100) surface (Δ*E*^≠^) was 244 kJ mol^−1^ at an octahedrally coordinated (six-coordinated) Al site (Fig. S8†). This value is much higher than 120 kJ mol^−1^ at a tetrahedrally coordinated Al center on a defective surface (Fig. 4a and S7†), corroborating that the CH_4_ activation on non-defective hydrated γ-Al_2_O_3_ (100) surfaces is kinetically less favorable than that on oxygen vacancy surfaces.

### Thermal stability and surface activation of templates for ideal 3D minimum-surface graphenes

Thus, TG, IR, and DFT calculations demonstrate that the initial desorption of H_2_O from γ-ANPs without CH_4_ (Fig. 3) cannot trigger off the reaction, while the subsequent elimination of surface oxygens with CH_4_ (Fig. 1e and f) is essential for generating the active surfaces in CH_4_-CVD. The resultant Lewis acid-base pair at oxygen vacancies can make the formation of single-walled NPG kinetically feasible with a significantly lower activation energy as compared to a radical mechanism.^24^ Interestingly, further desorption of H_2_O from the γ-ANP surfaces by heating from 900 to 1000 °C resulted in slower reaction rates (Table S1†). This could be explained by the structural changes of γ-ANP: The specific surface area of γ-ANPs decreased from 158 m^2^ g^−1^ for pristine to 139 m^2^ g^−1^ upon treatment at 900 °C for 2 h, and eventually decreased to 124 m^2^ g^−1^ upon annealing at 1000 °C for 2 h. The structural reorganization during CH_4_-CVD was also supported by ^27^Al nuclear magnetic resonance (NMR), which showed an increase in octahedrally coordinated stable Al centers (^[6]^Al) induced by annealing during CH_4_-CVD (Fig. S9†), and XRD (Fig. S10†).

Such structural reconstruction hinders the synthesis of three-dimensionally and periodically arranged single-walled graphene materials, even with the use of structurally ordered templates such as mesoporous silica^80^ and zeolites.^11^ Therefore, precise control of oxygen vacancies as well as high thermal stability of templates will be essential for CH_4_-CVD reactions on various ANPs (Fig. S11†) and other oxides.^20,23,72^ Further surface engineering, including activation by gaseous reductants, may be helpful in lowering the operation temperature for more efficient CH_4_ activation and NPG synthesis in the future.

## Conclusions

In summary, we have investigated the early-stage CH_4_ activation toward porous nanocarbon formation on γ-Al_2_O_3_ nanoparticles *via* reaction kinetics and surface analysis. We found that oxygen vacancies were formed on the surfaces of γ-Al_2_O_3_ nanoparticles upon their reaction with CH_4_ at temperatures higher than 800 °C. Carbon growth was promoted at the oxygen vacancies without the introduction of transition metal reaction centers. The initial dissociative adsorption of CH_4_ is the rate-limiting step because the overall rate of carbon growth is pseudo-first order for the CH_4_ partial pressure, and this is supported by DFT calculations. Surface Al at vacancy sites acts as a Lewis acid, whereas the adjacent surface oxygen acts as a Lewis base for dissociative adsorption to give surface-bound methyl and hydroxyl groups. This is followed by subsequent proton transfer to produce reactive surface-bound methylene species, leading to carbon growth. Carbon deposition from stable CH_4_ was faster on the surfaces of γ-ANPs with oxygen defects than on the deposited carbon films, and this makes it kinetically feasible to selectively form single-walled porous nanographene. Our work shows that precise surface engineering for introducing defects while enforcing the thermal stability of templates is crucial for accelerating CH_4_-CVD for better-quality NPG with fascinating features.^15,16^

## Data availability

All data associated with this study are available in the main text or the ESI.† Further data will be available upon request to the authors.

## Author contributions

M. Y., D. D. T., T. K. and K. Y. conceived and designed the project, and summarized all the data provided by co-authors. T. K. supervised the experiment part. S. G. contributed to thermogravimetry, mass spectrometry, and gas chromatography experiments. S. G. and H. N. contributed to the characterization of nanoporous carbon materials. M. Y., Y. G., M. T., and K. T. contributed to the *in situ* infrared spectroscopy in the presence of various gases. T. T. and T. Y. contributed to TEM and STEM of γ-ANPs. Q. Z. and A. A. conducted the quantum chemistry calculations. R. C. O. and D. D. T. supervised the quantum chemistry calculations and provided computational resources. M. Y., Q. Z., D. D. T., and K. Y. wrote the original draft, and contributed to visualization of the presented data. All authors contributed to review and editing.

## Conflicts of interest

There are no conflicts to declare.

## Supplementary Material

SC-013-D1SC06578E-s001
