# Supplementary material for: Porous nanographene formation on γ-alumina nanoparticles via transition-metal-free methane activation
Source: Chem Sci. 2022 Feb 22;13(11):3140–6. doi: 10.1039/d1sc06578e (PMC8926170; doi:10.1039/d1sc06578e)
Supplement: SC-013-D1SC06578E-s001 [file SC-013-D1SC06578E-s001.pdf]

Electronic Supplementary Material (ESI) for Chemical Science.
This journal is © The Royal Society of Chemistry 2022

Electronic Supplementary Information for

# Porous Nanographene Formation on γ-Alumina Nanoparticles *via* Transition-Metal-Free Methane Activation

Masanori Yamamoto,*[a] Qi Zhao,[c] Shunsuke Goto,[a] Yu Gu,[d‡] Takaaki Toriyama,[e] Tomokazu Yamamoto,[e] Hirotomo Nishihara,[a] Alex Aziz,[c] Rachel Crespo-Otero,[c] Devis Di Tommaso,*[c] Masazumi Tamura,[d‡] Keiichi Tomishige,[d] Takashi Kyotani[a] and Kaoru Yamazaki*[b†]

[a] *Institute of Multidisciplinary Research for Advanced Materials, Tohoku University, 2−1−1 Katahira, Aoba, Sendai 980−8577, Japan*
[b] *Institute for Materials Research, Tohoku University, 2−1−1 Katahira, Aoba, Sendai 980−8577, Japan*
[c] *Department of Chemistry, Queen Mary University of London, Mile End Road, London E1 4NS, UK*
[d] *Graduate School of Engineering, Tohoku University, 6−6−07 Aramaki, Aoba, Sendai 980−8579, Japan*
[e] *The Ultramicroscopy Research Center, Kyushu University, Motooka 744, Nishi, Fukuoka 819–0395, Japan*

[†] Present Address: *RIKEN Center for Advanced Photonics, RIKEN, 2-1 Hirosawa, Wako, Saitama 351–0198, Japan*

***To whom correspondence should be addressed**:
Dr. Masanori Yamamoto: yamamoto@mol-chem.com;
Dr. Devis Di Tommaso: d.ditommaso@qmul.ac.uk;
Dr. Kaoru Yamazaki: kaoru.yamazaki@riken.jp;

# Table of contents

S1. Materials and Methods
S2. Supplementary Figures, Tables, and Discussions
S3. Appendix. Optimized Structures in Quantum Chemistry Calculations

# S1. Materials and Methods

## S1.1 Sample

Methane ($CH_4$) was purchased from Sumitomo Seika Chemicals Co.,Ltd. with Pure grade (>99.0%) and SEG grade (>99.99%). Deuterated methane ($CD_4$, 99%-*d*) was purchased from Cambridge Isotope Laboratories, Inc. (DLM-144-PK, Lot: l-23094/E10089963). High purity γ-alumina nanoparticles (ANPs, TH 80/170; γ-$Al_2O_3$, particle size: ~10 nm, specific surface area: 158 $m^2$ $g^{-1}$) were donated from Sasol Limited. with Na (equivalent to $Na_2O$) = 20 ppm and Fe (equivalent to $Fe_2O_3$) = 50–100 ppm. All the chemicals were used as received unless otherwise noted.

## S1.2 Reaction Kinetics

Thermogravimetry was conducted on a thermogravimeter (Netzsch, STA 2500 *Regulus*) under a steady flow of He with various concentration of $CH_4$ at the total flow rate of 100 mL $min^{-1}$. The standard reactor volume was 50 mL. To suppress air contamination during the analysis, the thermogravimeter was surrounded by a chamber filled with Ar gas supplied at a flow rate of 1 L $min^{-1}$. Typically, 32 mg of γ-ANPs was used for the kinetic analysis of $CH_4$-CVD. The samples were loaded on the reactor of the thermogravimeter, and first heated from room temperature to a specified temperature (1128 – 1173 K) at 10 K $min^{-1}$ under a steady flow of He. This was followed by the constant-temperature heating at the specified temperature for 30 min under a steady flow of He. After the pretreatment, $CH_4$ was introduced to the reactor with a specified partial pressure to initiate $CH_4$-CVD. The rate of $CH_4$-CVD for the kinetic analysis of the first-layer deposition was determined at an inflection point. Formation of nanoporous graphene was confirmed by x-ray diffraction and Raman spectroscopy (Figure S1).

## S1.3 Characterization of γ-$Al_2O_3$ Nanoparticles

High resolution annular dark-field scanning transmission electron microscopy (ADF-STEM) images were captured using JEM-ARM200-F (JEOL Ltd, Japan) at an accelerating voltage of 200kV.

*In situ* infrared (IR) spectra of the $Al_2O_3$ nanoparticles (ca. 18.0 mg) at the specified temperature were recorded on a Nicolet 6700 FT-IR spectrometer (ThermoScientific) with a diffuse reflectance infrared Fourier transform (DRIFT) method under a steady flow of Ar at 30 mL $min^{-1}$. The intensity was reported as the Kubelka-Munk function.[S1] The temperature of the sample during the *in-situ* IR measurements was monitored by using an infrared thermometer (Keyence, FT-H40K) to ensure the temperature. We performed 120 scans.

Temperature programmed desorption (TPD) was measured on a gas chromatogram (GC, Varian 490-GC, GL Science). Approximately 1 g of γ-ANP was gently packed in a quartz reactor tube with stacked height of 3~4 cm using quartz wool (4-9 μm, Toso Company, Ltd.). The reactor tube was heated at the rate of 10 K $min^{-1}$ under a steady flow of He (200 mL $min^{-1}$), and the evolved $H_2O$ was analyzed by GC. The sampling interval was approximately 2.5 min.

TPD of $H_2O$ was immediately followed by TPD of $NH_3$ with the same configuration of experiment. All the processes are under a steady flow at a total flow rate of 200 mL $min^{-1}$. After cooling to r.t. under He, a mixture of 1.0% of $NH_3$ and 99% of He was introduced to the quartz reactor tube for 30 min. Then, the reactor tube was subjected to a steady flow of 100% He for 60 min to remove off the gas-phase $NH_3$. The reactor tube was then heated at the rate of 5 K $min^{-1}$ under a steady flow of He, and the desorbed $NH_3$ was analyzed by GC.

Magic-angle-spinning (MAS) $^{27}Al$ nuclear magnetic resonance (NMR) of solid samples were recorded on a JEOL ECA800 (208 MHz) spectrometer. Samples were spun at 25 kHz. The data acquisition employed short radiofrequency pulses (18º flip angle) with relaxation delays of 10 s. The chemical shifts have been reported in δ ppm units with reference to an external standard of a 1 M aqueous solution of aluminium nitrate ($Al(NO_3)_3$, 0.00 ppm).

## S1.4 Quantum Chemistry Calculations

Density functional theory (DFT) calculations on γ-$Al_2O_3$ surfaces with methane were performed by using the "Vienna *ab initio* simulation package" (VASP, version 6.1.1).[S2] We performed geometry optimization of local minima (EQs) and transition states (TSs) with the Perdew–Burke–Ernzerhof (PBE) generalized-gradient approximation (GGA) for the exchange and correlation terms,[S3] together with the Grimme's-D3 dispersion correction.[S4] A plane-wave basis set was employed within the framework of the projector augmented wave method.[S5,S6] The plane-wave cutoff was 450 eV. K-point mesh ware sampled by the (3×3×1) Monkhorst-Pack *k*-point sampling scheme. We used the threshold of self-consistent field energy calculation of $1.0 \times 10^{-6}$ eV $atom^{-1}$.

The surface of γ-$Al_2O_3$ was modeled using a supercell approach with periodic boundary conditions. To generate the surface models, we first optimized the internal coordinates and cell parameters of the bulk structure of γ-$Al_2O_3$. As a starting point, we used the coordinates of γ-$Al_2O_3$ ($P2_1/m$ space group).[S9] The optimized lattice parameters of γ-$Al_2O_3$ were found to be $a$ = 5.538 Å, $b$ = 8.347 Å, $c$ = 8.024 Å, $\beta$ = 90.60 deg and $\alpha = \gamma$ = 90.00 deg, in good agreement with previous DFT calculations using the dual-range local meta-GGA ML-11 functional and the meta non-separable gradient approximation MN12-L functionals.[S7] Starting from the optimized structure, we constructed a partially hydrated γ-$Al_2O_3$ (100) surface with an oxygen vacancy as a surface slab containing 96 atoms (corresponding to 4 atomic layers) with a thickness of 12 Å and a vacuum of 15 Å. The initial surface hydration was 7.1 μmol $m^{-2}$. During the structural optimization for γ-$Al_2O_3$, the all atoms were relaxed. The structure of the triclinic surface unit cell was $a$ = 8.071 Å, $b$ = 8.404 Å, $c$ = 26.383 Å, and $\alpha = \beta = \gamma$ = 90.00 deg.

TSs for the elementary reactions were located by using the climbing image nudged elastic band (CI-NEB) method.[S8,S9] The convergence of forces for the geometry optimizations of both EQs and TSs were set to be 0.03 eV $Å^{-1}$. All optimized structures are collected in S3 according to the POSCAR format for VASP.

The VASPKIT was used to deal with the charge density and the spin density.[S10]Atomistic models of alumina surfaces were constructed according to the results of ADF-STEM, TPD, and IR analysis of γ-ANPs. Previous DFT calculations suggest that (100) and (110) surfaces are the most stable surface of γ-$Al_2O_3$,[S11] and indeed the high-resolution ADF-STEM of γ-ANPs (Figure 2) shows the presence of {100} surfaces. Significant desorption of water upon heating [S11,S12] was confirmed by TPD (Figure 3b), while isolated hydroxyl groups at around 3701 $cm^{-1}$ were confirmed by IR (Figure 3a) to remain even after annealing at 900 ºC for 30

min. Indeed, introduction of hydroxyl groups on surface models gave both thermodynamically and kinetically favored process in $CH_4$ activation on surfaces of $\gamma$-$Al_2O_3$.[S12,13]

# S2. Supplementary Figures, Tables, and Discussions

## S2.1 Characterization of Nano-Porous Graphene (**NPG**)

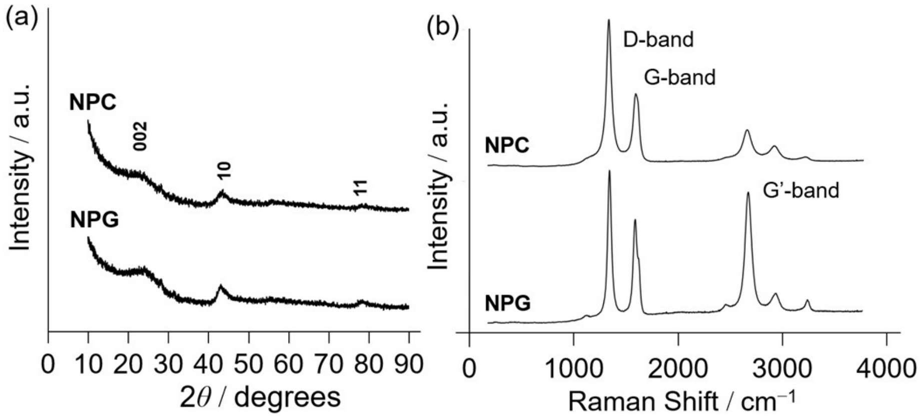


***Figure S1.*** (a) XRD patterns and (b) Raman spectra of nano-porous carbon (**NPC**) and nano-porous graphene (**NPG**).

**NPG** was prepared and characterized by $N_2$ physisorption, XRD, and Raman spectroscopy. The specific surface area of **NPC** was determined by $N_2$ physisorption and the subsequent BET analysis to be $2.3 \times 10^3$ $m^2$ $g^{-1}$, while **NPG** gave $1.8 \times 10^3$ $m^2$ $g^{-1}$. These values are well approaching to an ideal value for 2-dimensional graphene (2627 $m^2$ $g^{-1}$). Single-layered deposition by $CH_4$-CVD with a specified reaction time was also confirmed by TEM.[S14] Both XRD and Raman showed single-walled nature of **NPG**: The suppressed 002 peaks at $2\theta = 22^o$ for carbons indicates the fewer stacking of graphene layer, while sharp peaks for 10 at $2\theta = 43^o$ indicates the successful growth of hexagons (nanographene) developed in a 2D plane.[S14] Raman spectra of **NPG** showed an intensified and red-shifted G'-band, supporting the growth of single-walled graphene structures[S14-S16] in **NPG** after annealing.

## S2.2 Reductive treatment of ANP by $H_2$ before $CH_4$-CVD

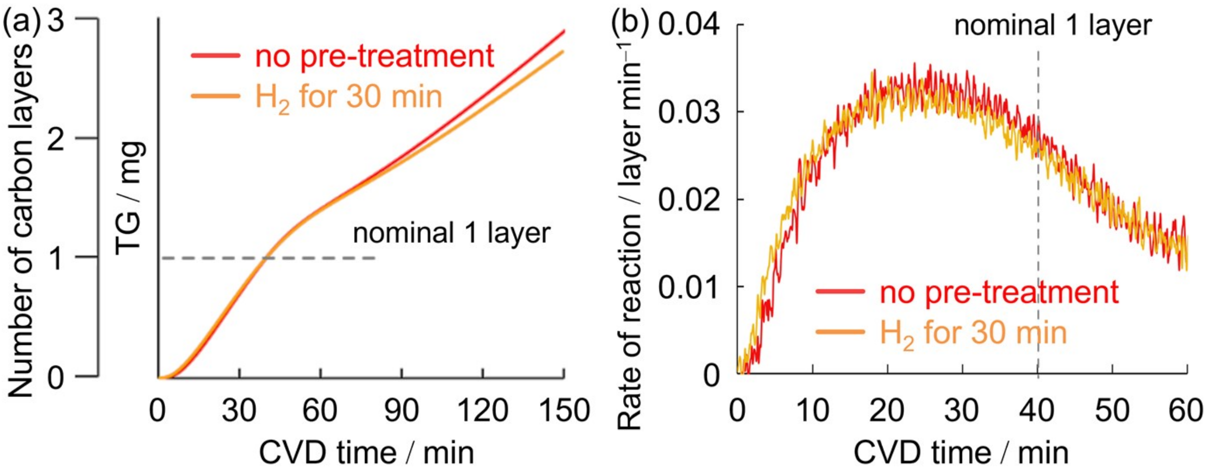


***Figure S2.*** Kinetic analysis of $CH_4$-CVD for porous nanographene with or without pre-treatment of $H_2$ gas before $CH_4$-CVD. For the control experiment, He was introduced instead of $H_2$ for the same period. (a) Weight changes during $CH_4$-CVD at 900°C as monitored by TG. $CH_4$ was introduced to the reactor at 0 min. (b) The rate of reaction for $CH_4$-CVD.

## S2.3 Surface Characterization by STEM

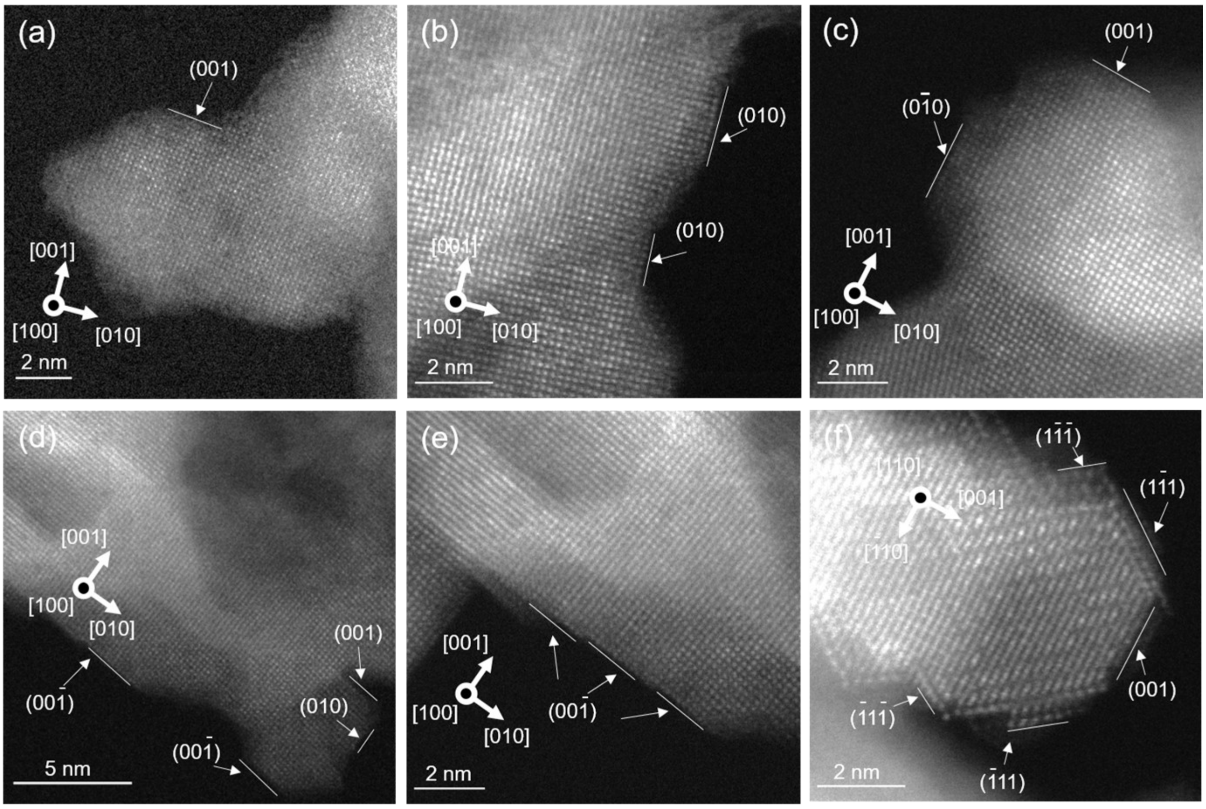


***Figure S3.*** High resolution STEM-ADF image of γ-ANPs. (a–e) [100] orientation, and (f) [110] orientation.

## S2.4 Stability of Surface OH groups toward Surface Activation

In order to analyze the stability of the OH groups on the γ-ANP surface for the surface activation, we performed the *in-situ* IR experiments under a steady flow of $CD_4$. We find that almost all protons are labile in the presence of $CD_4$ at temperatures higher than 600 ºC (Figures S4, S5) while the structure of bulk region remained almost unchanged during the $CH_4$-CVD according to the $^{27}$Al NMR spectra (Figure S9). The D-H exchange between $CD_4$ and isolated OH groups occurred on the γ-ANP surface above 600 ºC, and the OH stretching band at $\nu_{OH}$ = 3701 cm$^{-1}$ depressed with time constants of 1.2 min and the OD stretching band at $\nu_{OD}$ = 2730 cm$^{-1}$ evolved as shown in Figure S4.

This isotope shift can be quantitively rationalized by the change of the reduced mass $\mu$ by the H-D exchange. The vibration frequency $\nu$ of the OH stretching mode is described in eq. (S1) under the harmonic approximation,

$$\nu = \frac{1}{2\pi}\sqrt{\frac{k_f}{\mu}} = \frac{1}{2\pi}\sqrt{\frac{k_f}{\frac{m_O m_H}{m_O + m_H}}}, \qquad \text{(S1)}$$

where $m_O$ = 16 amu, and $m_H$ = 1 amu are the masses of oxygen and hydrogen atoms, respectively. The effect of isotope exchange on the vibrational force constant $k_f$ is negligible and the frequency of the deuterated system $\nu_{OD}$ can be written as the rate between reduced mass of OD group $\mu_{OD}$ and that of OH $\mu_{OH,}$, and the frequency of the original system $\nu_{OH}$ = 3701 cm$^{-1}$,

$$\nu_{OD} = \frac{\sqrt{\mu_{OD}}}{\sqrt{\mu_{OH}}}\nu_{OH} = \frac{\sqrt{\frac{m_O m_D}{m_O + m_D}}}{\sqrt{\frac{m_O m_H}{m_O + m_H}}}\nu_{OH}, \qquad \text{(S2)}$$

where $m_D$ = 2 amu is the mass of deuterium. Resultant $\nu_{OD}$ is calculated to be 2693 cm$^{-1}$, which qualitatively agrees with the experimental value (2730 cm$^{-1}$).

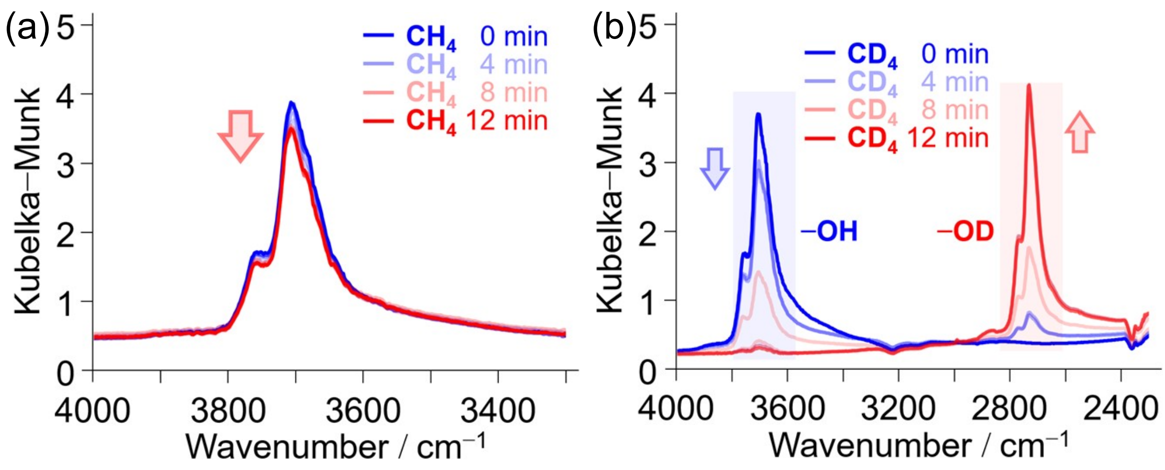

***Figure S4.*** Temporal profiles of the OH stretching bands in IR spectra of ANPs at 900 ºC (a) in the presence of $CH_4$ (2 mL min$^{-1}$) and (b) in the presence of $CD_4$ (2 mL min$^{-1}$). Depletion at 2350 cm$^{-1}$ is due to $CO_2$.

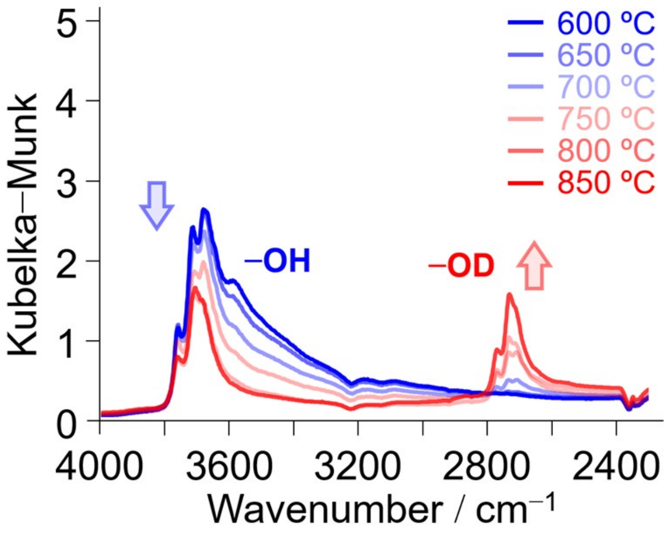


***Figure S5.*** Time-course of IR spectra of γ-ANPs in the presence of $CD_4$ at the elevation rate of 16.7 K min$^{-1}$ from 600 ºC to 850 ºC. Depletion at 2350 cm$^{-1}$ is due to $CO_2$.

## S2.5 Stability of Oxygen Vacancy Sites and Reactivity of $CH_4$ on Them

***Table S1.*** Summary of $H_2O$/$NH_3$ TPD[a] and $CH_4$-CVD.

| Conditions for pre-activation[b] | Evolved gas | | Rate of reactions[d] |
|---|---|---|---|
| | $H_2O$[c] | $NH_3$ | |
| 700 ºC for 30 min | 1.1 mmol g$^{-1}$ | 33 μmol g$^{-1}$ | --- |
| 900 ºC for 30 min | 1.4 mmol g$^{-1}$ | 19 μmol g$^{-1}$ | 4.8 × 10$^{-9}$ mol s$^{-1}$ |
| 1000 ºC for 30 min | 1.5 mmol g$^{-1}$ | 21 μmol g$^{-1}$ | 3.6 × 10$^{-9}$ mol s$^{-1}$ |

[a] The details are shown in the section S1.3; [b] under a steady flow of He; [c] The amount of water desorbed at the temperatures higher than 300 ºC; [d] The rate for the first-layer deposition under the standard $CH_4$-CVD condition at 900 ºC with a steady flow of $CH_4$ (20 mL min$^{-1}$).

S2.6 Density Functional Chemical Calculations

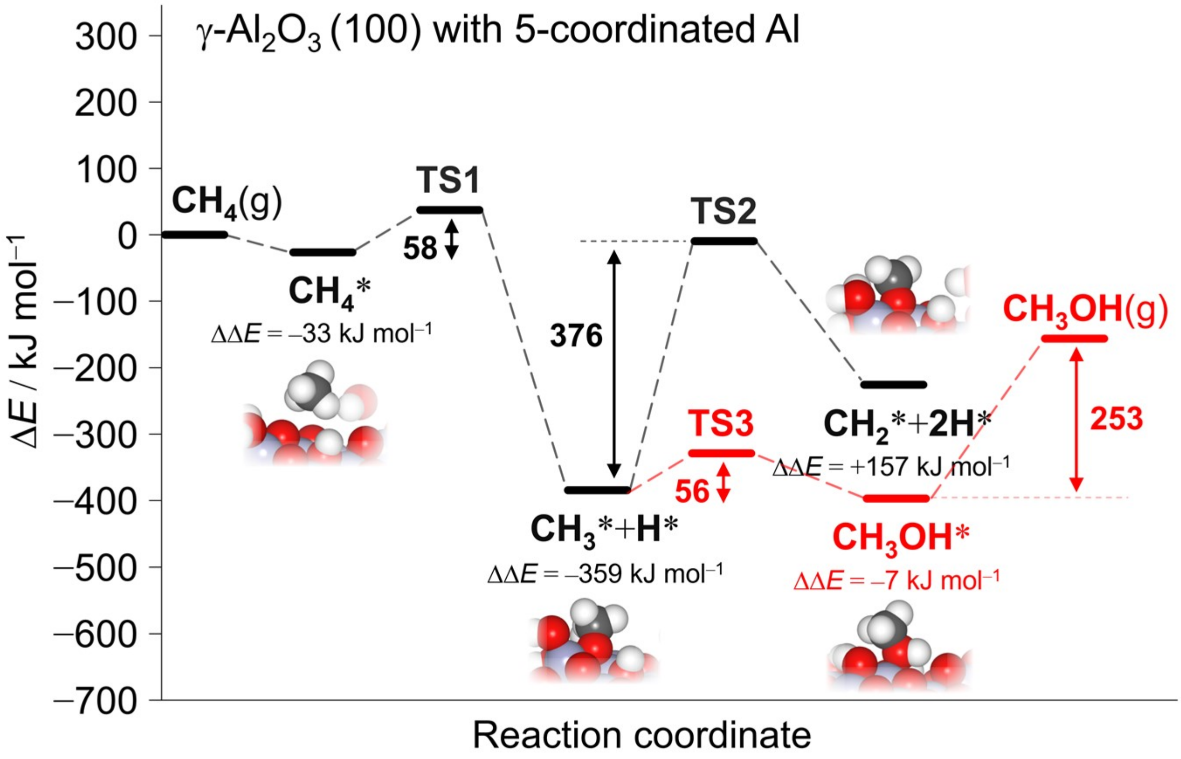


***Figure S6.*** Black line: Energy profile for the formation of a $CH_4$ σ complex and the subsequent C–H bond cleavage on a γ-$Al_2O_3$ (100) surface. Red line: Conversion of $CH_4$ to $CH_3OH$ on a γ-$Al_2O_3$ (100) surface. The reactive site is a 5-coordinated-Al.

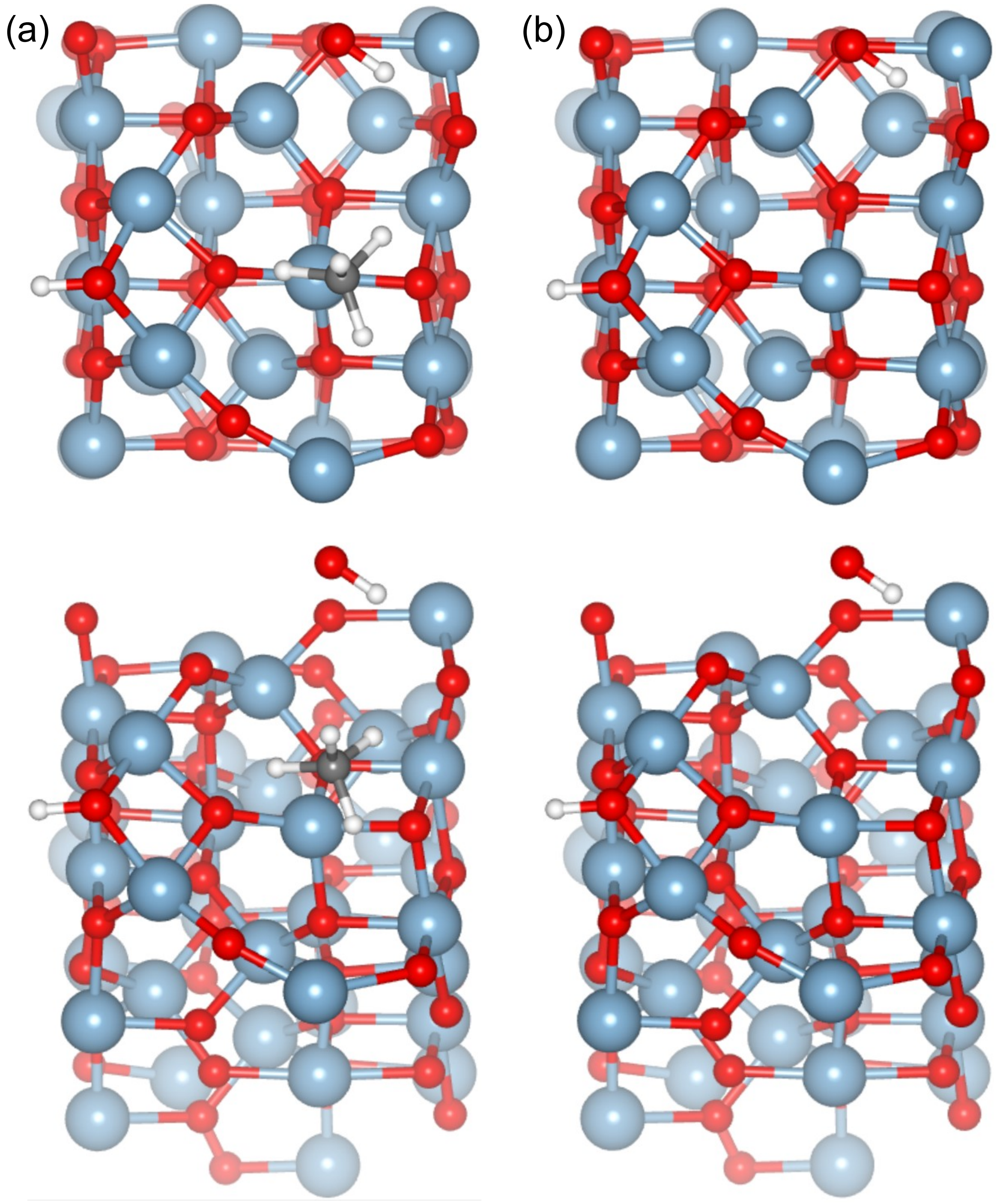


***Figure S7.*** (a) Geometry of a γ-$Al_2O_3$ (100) surface with an adsorbed $CH_4$ and (b) the same geometry without $CH_4$ for clarity. Red: oxygen, Blue; aluminum, gray: carbon, and white: hydrogen atoms. The reactive site is tetrahedrally-coordinated (4-coordinated) Al center, and the coordinate is the same for $CH_4$* in Figure 4a of the manuscript. The coordinates are shown in Section S3.

**Table S2.** Calculated Bader charges *q* (in units of |*e*|) of adsorbed $\mathbf{CH_4}$* and $\mathbf{CH_3}$* species on a γ-$Al_2O_3$ (100) surface associated with the $CH_4 \rightarrow CH_3 + H$ elementary step. The value in brackets indicates the formal oxidation state corresponding to the Bader charge.

| **Property** | **Initial State** | **Transition State (TS1)** | **Final State** |
|---|---|---|---|
| q($CH_4$*) | –0.005 | --- | --- |
| q($CH_3$*) | --- | –0.65 | –0.61 |
| q(C) | –0.16 | –0.77 | –0.71 |
| q(H3*des) | +0.071 | +0.66 | +0.66 |
| q(Al7) | +2.43 (+3) | +2.35 (+3) | +2.35 (+3) |
| q(O23) | –1.58 (–2) | –1.49 (–2) | –1.59 (–2) |

**Table S3.** Calculated Bader charges *q* (in units of |*e*|) of adsorbed $\mathbf{CH_3}$* and $\mathbf{CH_2}$* species on a γ-$Al_2O_3$ (100) surface associated with the $CH_3 \rightarrow CH_2 + H$ elementary step. The value in brackets indicates the formal oxidation state corresponding to the Bader charge.

| **Property** | **Initial State** | **Transition State (TS2)** | **Final State** |
|---|---|---|---|
| q($CH_3$*) | –0.61 | --- | --- |
| q($CH_2$*) | --- | –0.71 | –0.71 |
| q(C) | –0.71 | –0.89 | –0.80 |
| q(H5*des) | +0.06 | +0.64 | +0.67 |
| q(Al7) | +2.35 (+3) | +2.35 (+3) | +2.38 (+3) |
| q(O45) | –1.63 (–2) | –1.50 (–2) | –1.57 (–2) |

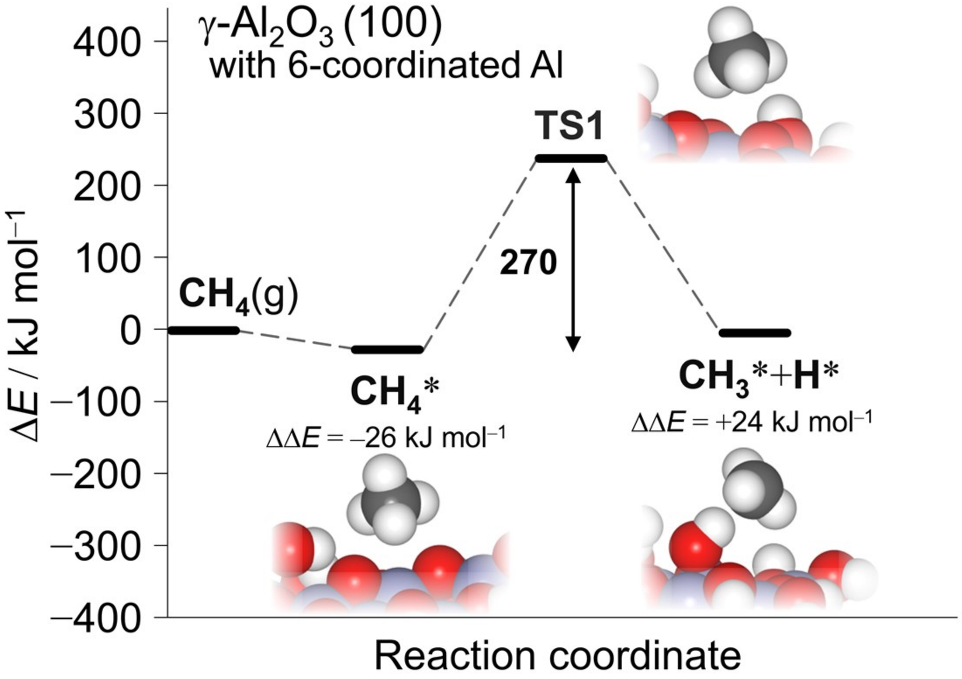


***Figure S8.*** Energy profile for the formation of a $CH_4$ σ complex and the subsequent C–H bond cleavage on a γ-$Al_2O_3$ (100) surface with no oxygen defect. The reactive site is an octahedrally-coordinated (6-coordinated) Al center, and the clouded surface gives radical mechanism rather than the Lewis acid-base mechanism for the bond cleavage reaction.

S2.7 MAS $^{27}$Al NMR

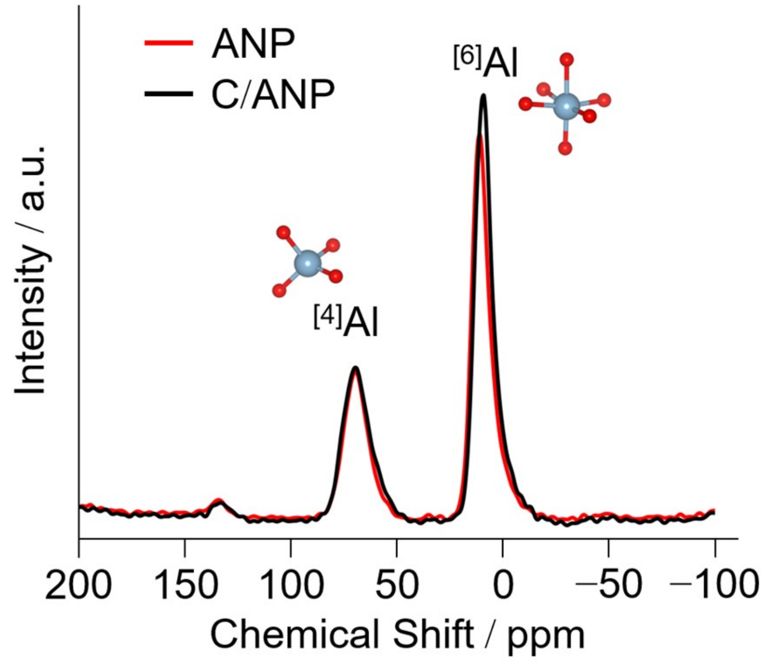


***Figure S9.*** Normalized MAS $^{27}$Al NMR of γ-ANPs before and after $CH_4$-CVD. Relative intensity of the peak for octahedrally coordinated Al-center ($^{[6]}$Al) in the up-field (δ = 9 ppm)[S17] was enhanced as compared with that for tetrahedrally coordinated Al-center ($^{[4]}$Al) in the down-field (δ = 68 ppm)[S17] after $CH_4$-CVD.

S2.8 Structural Analysis of ANPs by XRD

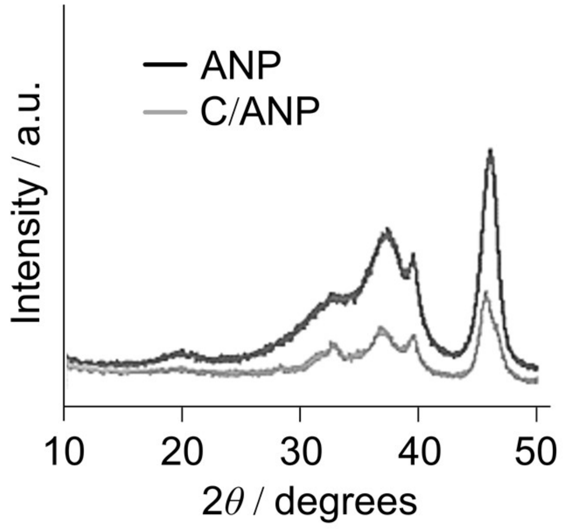


***Figure S10.*** XRD of γ-ANPs before (ANP) and after $CH_4$-CVD (C/ANP).

## S2.9 Crystal Structure Dependency on $CH_4$-CVD

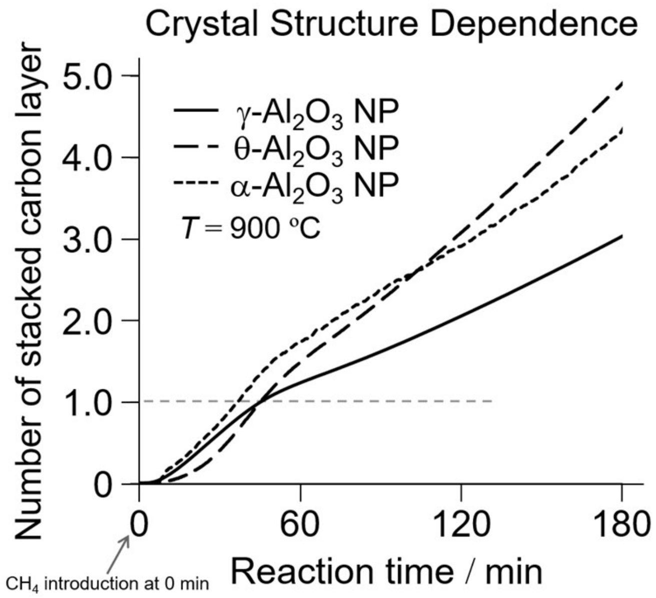


***Figure S11.*** Time-course of weight changes during $CH_4$-CVD on various crystal structures of $Al_2O_3$ at 900 ºC as monitored by TG.

Curvature reflecting the difference in the rates of $CH_4$-CVD reactions was recognized at nearly single-layered deposition of carbon (Figs. 1a and 1b), but there was exception at higher partial pressure of $CH_4$ or at lower temperatures showing the curvature at the number of carbon layers > 1. We also noticed such exceptions for θ-ANP and α-ANP (Fig. S11). This could indicate that a mixture growth on the $Al_2O_3$ nanoparticles (first layer) and on carbon layer (second layer) was significant under these conditions. Thus, lower partial pressure of $CH_4$ and higher reaction temperatures as well as the use of γ-ANP would be important for single-layered carbon deposition.

## References


S1. P. Kubelka, *Part. J. Opt. Soc. Am.* **1948**, *38*, 448.

S2. G. Kresse, J. Furthmüller, *Comput. Mater. Sci.* **1996**, *6*, 15–50.

S3. J. P. Perdew, K. Burke, M. Ernzerhof, *Phys. Rev. Lett.* **1996**, *77*, 3865–3868.

S4. S. Grimme, J. Antony, S. Ehrlich, H. Krieg, *J. Chem. Phys.* **2010**, *132*, 154104.

S5. P. E. Blöchl, *Phys. Rev. B* **1994**, *50*, 17953–17979.

S6. G. Kresse, D. Joubert, *Phys. Rev. B* **1999**, *59*, 1758–1775.

S7. G. Henkelman, H. Jónsson, *J. Chem. Phys.* **2000**, *113*, 9978–9985.

S8. G. Henkelman, B. P. Uberuaga, H. Jónsson, *J. Chem. Phys.* **2000**, *113*, 9901–9904.

S9. J. Gu, J. Wang, J. Leszczynski, *ACS Omega* **2018**, *3*, 1881–1888.

S10. V. Wang, N. Xu, J.C. Liu, G. Tang, W.T. Geng, VASPKIT: A User-Friendly Interface Facilitating High-Throughput Computing and Analysis Using VASP Code, arXiv:1908.08269

S11. a) M. Digne, P. Sautet, P. Raybaud, P. Euzen, H. Toulhoat, *J. Catal.* **2002**, *211*, 1–5; b) M. Digne, P. Sautet, P. Raybaud, P. Euzen, H. Toulhoat, *J. Catal.* **2004**, *226*, 54–68.

S12. R. Wischert, C. Copéret, F. Delbecq, P. Sautet, *Angew. Chem. Int. Ed.* **2011**, *50*, 3202–3205.

S13. R. Wischert, P. Laurent, C. Copéret, F. Delbecq, P. Sautet, *J. Am. Chem. Soc.* **2012**, *134*, 14430–14449.

S14. M. Yamamoto, S. Goto, R. Tang, Y. Hayasaka, Y. Yoshioka, M. Ito, M. Morooka, H. Nishihara, T. Kyotani, *ACS Appl. Mater. Interfaces* **2021**, *13*, 38613–38622.

S15. A. C. Ferrari, J. C. Meyer, V. Scardaci, C. Casiraghi, M. Lazzeri, F. Mauri, S. Piscanec, D. Jiang, K. S. Novoselov, S. Roth, A. K. Geim, *Phys. Rev. Lett.* **2006**, 97, 187401.

S16. A. Gupta, G. Chen, P. Joshi, S. Tadigadapa, P.C. Eklund, *Nano Lett.* **2006**, *6*, 2667–2673.

S17. L. Samain, A. Jaworski, M. Edén, D. M. Ladd, D.-K. Seo, F. J. Garcia-Garcia, U. Häussermann, *J. Solid State Chem.* **2014**, *217*, 1–8.

# S3. Appendix: Optimized Structures and Energies in Quantum Chemistry Calculations

## S3.1 Optimized Structures and Energies for Figure 4

**Coordinate** for a γ-$Al_2O_3$ (100) surface with $CH_4$ σ-complex with a surface proton density of 7.1 μmol $m^{-2}$ at a 4-coordinated Al site.
Spin Multiplicity <$S^2$>: 0.006
Absolute Energy: –719.479682 eV.

| | |
|---|---|
| cell_length_a | 8.07075 |
| cell_length_b | 8.40443 |
| cell_length_c | 26.38316 |
| cell_angle_alpha | 90 |
| cell_angle_beta | 90 |
| cell_angle_gamma | 90 |
| symmetry_space_group_name_H-M | 'P 1' |
| symmetry_Int_Tables_number | 1 |

| | | | | | |
|---|---|---|---|---|---|
| H1 | 1.0 | 0.012525 | 0.411272 | 0.458504 | H |
| H2 | 1.0 | 0.389156 | 0.758581 | 0.967927 | H |
| H3 | 1.0 | 0.698026 | 0.308049 | 0.531916 | H |
| H4 | 1.0 | 0.739746 | 0.514269 | 0.518631 | H |
| H5 | 1.0 | 0.530180 | 0.443189 | 0.520374 | H |
| H6 | 1.0 | 0.646493 | 0.458356 | 0.577451 | H |
| H7 | 1.0 | 0.865656 | 0.323823 | 0.970992 | H |
| H8 | 1.0 | 0.746757 | 0.853057 | 0.493596 | H |
| C1 | 1.0 | 0.654231 | 0.430645 | 0.536903 | C |
| O1 | 1.0 | 0.110810 | 0.590287 | 0.157363 | O |
| O2 | 1.0 | 0.126991 | 0.579497 | 0.367156 | O |
| O3 | 1.0 | 0.616791 | 0.907808 | 0.048522 | O |
| O4 | 1.0 | 0.618926 | 0.917007 | 0.260722 | O |
| O5 | 1.0 | 0.844216 | 0.414243 | 0.993966 | O |
| O6 | 1.0 | 0.863210 | 0.412263 | 0.207681 | O |
| O7 | 1.0 | 0.830444 | 0.416952 | 0.419161 | O |
| O8 | 1.0 | 0.368215 | 0.102009 | 0.097134 | O |
| O9 | 1.0 | 0.363299 | 0.085661 | 0.312928 | O |
| O10 | 1.0 | 0.910452 | 0.097248 | 0.108539 | O |
| O11 | 1.0 | 0.901999 | 0.110408 | 0.320372 | O |
| O12 | 1.0 | 0.418851 | 0.425306 | 0.999761 | O |
| O13 | 1.0 | 0.396402 | 0.420826 | 0.210593 | O |
| O14 | 1.0 | 0.401961 | 0.441531 | 0.423833 | O |
| O15 | 1.0 | 0.159017 | 0.913791 | 0.060543 | O |
| O16 | 1.0 | 0.152386 | 0.907277 | 0.268392 | O |
| O17 | 1.0 | 0.644021 | 0.580040 | 0.158151 | O |
| O18 | 1.0 | 0.638591 | 0.601353 | 0.369087 | O |
| O19 | 1.0 | 0.907293 | 0.404002 | 0.107473 | O |
| O20 | 1.0 | 0.908008 | 0.411988 | 0.322313 | O |
| O21 | 1.0 | 0.421249 | 0.085696 | 0.000782 | O |
| O22 | 1.0 | 0.396766 | 0.084955 | 0.212754 | O |
| O23 | 1.0 | 0.426853 | 0.132654 | 0.444146 | O |
| O24 | 1.0 | 0.156904 | 0.597636 | 0.059375 | O |
| O25 | 1.0 | 0.156913 | 0.596491 | 0.269328 | O |
| O26 | 1.0 | 0.643976 | 0.920901 | 0.159718 | O |
| O27 | 1.0 | 0.646395 | 0.916486 | 0.382605 | O |
| O28 | 1.0 | 0.112524 | 0.911602 | 0.158270 | O |
| O29 | 1.0 | 0.103110 | 0.923428 | 0.366921 | O |
| O30 | 1.0 | 0.617146 | 0.603980 | 0.047211 | O |
| O31 | 1.0 | 0.618472 | 0.589456 | 0.258414 | O |
| O32 | 1.0 | 0.845170 | 0.118494 | 0.998522 | O |
| O33 | 1.0 | 0.863477 | 0.090727 | 0.207279 | O |
| O34 | 1.0 | 0.845781 | 0.093978 | 0.425296 | O |

| | | | | | |
|---|---|---|---|---|---|
| O35 | 1.0 | 0.366314 | 0.408812 | 0.095626 | O |
| O36 | 1.0 | 0.369713 | 0.412090 | 0.310746 | O |
| O37 | 1.0 | 0.137828 | 0.253004 | 0.157850 | O |
| O38 | 1.0 | 0.142084 | 0.251362 | 0.371803 | O |
| O39 | 1.0 | 0.638516 | 0.258524 | 0.057742 | O |
| O40 | 1.0 | 0.647124 | 0.253117 | 0.260716 | O |
| O41 | 1.0 | 0.891289 | 0.752840 | 0.109178 | O |
| O42 | 1.0 | 0.894881 | 0.740346 | 0.320221 | O |
| O43 | 1.0 | 0.330572 | 0.756593 | 0.000109 | O |
| O44 | 1.0 | 0.363575 | 0.752308 | 0.213126 | O |
| O45 | 1.0 | 0.354565 | 0.755493 | 0.425107 | O |
| O46 | 1.0 | 0.900661 | 0.757970 | 0.999763 | O |
| O47 | 1.0 | 0.894859 | 0.750823 | 0.210337 | O |
| O48 | 1.0 | 0.914227 | 0.730620 | 0.424643 | O |
| O49 | 1.0 | 0.383665 | 0.753497 | 0.104142 | O |
| O50 | 1.0 | 0.382431 | 0.755530 | 0.315846 | O |
| O51 | 1.0 | 0.122889 | 0.256524 | 0.053850 | O |
| O52 | 1.0 | 0.114393 | 0.256781 | 0.266551 | O |
| O53 | 1.0 | 0.645985 | 0.251255 | 0.163526 | O |
| O54 | 1.0 | 0.633320 | 0.260363 | 0.366448 | O |
| O55 | 1.0 | 0.135368 | 0.417876 | 0.462171 | O |
| O56 | 1.0 | 0.648143 | 0.919690 | 0.495631 | O |
| Al1 | 1.0 | 0.394203 | 0.925716 | 0.056171 | Al |
| Al2 | 1.0 | 0.388366 | 0.925054 | 0.268298 | Al |
| Al3 | 1.0 | 0.137549 | 0.424010 | 0.111684 | Al |
| Al4 | 1.0 | 0.141246 | 0.417788 | 0.321118 | Al |
| Al5 | 1.0 | 0.637737 | 0.429274 | 0.013999 | Al |
| Al6 | 1.0 | 0.622752 | 0.427441 | 0.213397 | Al |
| Al7 | 1.0 | 0.608664 | 0.430060 | 0.405948 | Al |
| Al8 | 1.0 | 0.870145 | 0.920810 | 0.162533 | Al |
| Al9 | 1.0 | 0.887620 | 0.909823 | 0.392393 | Al |
| Al10 | 1.0 | 0.643600 | 0.080089 | 0.013552 | Al |
| Al11 | 1.0 | 0.625514 | 0.075905 | 0.214252 | Al |
| Al12 | 1.0 | 0.616620 | 0.031576 | 0.440742 | Al |
| Al13 | 1.0 | 0.869725 | 0.582574 | 0.161939 | Al |
| Al14 | 1.0 | 0.870383 | 0.590931 | 0.376095 | Al |
| Al15 | 1.0 | 0.392237 | 0.586595 | 0.055100 | Al |
| Al16 | 1.0 | 0.392264 | 0.581774 | 0.269067 | Al |
| Al17 | 1.0 | 0.141404 | 0.079642 | 0.113867 | Al |
| Al18 | 1.0 | 0.132394 | 0.082742 | 0.323974 | Al |
| Al19 | 1.0 | 0.896764 | 0.243816 | 0.053385 | Al |
| Al20 | 1.0 | 0.880726 | 0.261485 | 0.272586 | Al |
| Al21 | 1.0 | 0.506431 | 0.257764 | 0.115979 | Al |
| Al22 | 1.0 | 0.502352 | 0.244985 | 0.315937 | Al |
| Al23 | 1.0 | 0.883448 | 0.251325 | 0.161685 | Al |
| Al24 | 1.0 | 0.864478 | 0.244070 | 0.377086 | Al |
| Al25 | 1.0 | 0.102397 | 0.758243 | 0.015780 | Al |
| Al26 | 1.0 | 0.130648 | 0.750868 | 0.221422 | Al |
| Al27 | 1.0 | 0.233455 | 0.589466 | 0.428129 | Al |
| Al28 | 1.0 | 0.514930 | 0.750651 | 0.164319 | Al |
| Al29 | 1.0 | 0.500458 | 0.764329 | 0.377473 | Al |
| Al30 | 1.0 | 0.139321 | 0.752232 | 0.113593 | Al |
| Al31 | 1.0 | 0.130852 | 0.757320 | 0.325584 | Al |
| Al32 | 1.0 | 0.769038 | 0.756627 | 0.052245 | Al |
| Al33 | 1.0 | 0.753830 | 0.752164 | 0.270819 | Al |
| Al34 | 1.0 | 0.325129 | 0.255271 | 0.029516 | Al |
| Al35 | 1.0 | 0.266034 | 0.253710 | 0.218426 | Al |
| Al36 | 1.0 | 0.275653 | 0.267166 | 0.429342 | Al |

**Coordinate** for a calculated transition state of the initial $CH_4$ activation on a γ-$Al_2O_3$ (100) surface (**TS1**) in Figure 4.
Spin Multiplicity <$S^2$>: 0.023
Absolute Energy: –718.02755056 eV.

| | |
|---|---|
| cell_length_a | 8.07075 |
| cell_length_b | 8.40443 |
| cell_length_c | 26.38316 |
| cell_angle_alpha | 90 |
| cell_angle_beta | 90 |
| cell_angle_gamma | 90 |
| symmetry_space_group_name_H-M | 'P 1' |
| symmetry_Int_Tables_number | 1 |

| | | | | | |
|---|---|---|---|---|---|
| H1 | 1.0 | 0.999497 | 0.303556 | 0.474501 | H |
| H2 | 1.0 | 0.391601 | 0.760793 | 0.967337 | H |
| H3 | 1.0 | 0.464363 | 0.203673 | 0.481020 | H |
| H4 | 1.0 | 0.712861 | 0.509912 | 0.506832 | H |
| H5 | 1.0 | 0.497569 | 0.470945 | 0.516232 | H |
| H6 | 1.0 | 0.648007 | 0.314327 | 0.518546 | H |
| H7 | 1.0 | 0.867957 | 0.324562 | 0.970619 | H |
| H8 | 1.0 | 0.748970 | 0.925398 | 0.510760 | H |
| C1 | 1.0 | 0.612045 | 0.425223 | 0.498948 | C |
| O1 | 1.0 | 0.113042 | 0.593115 | 0.156824 | O |
| O2 | 1.0 | 0.126718 | 0.578692 | 0.361392 | O |
| O3 | 1.0 | 0.619109 | 0.909381 | 0.048429 | O |
| O4 | 1.0 | 0.622889 | 0.918380 | 0.260880 | O |
| O5 | 1.0 | 0.846619 | 0.415135 | 0.993565 | O |
| O6 | 1.0 | 0.865476 | 0.413679 | 0.207422 | O |
| O7 | 1.0 | 0.851724 | 0.406386 | 0.417653 | O |
| O8 | 1.0 | 0.370901 | 0.103750 | 0.097220 | O |
| O9 | 1.0 | 0.368545 | 0.085289 | 0.314086 | O |
| O10 | 1.0 | 0.913061 | 0.098563 | 0.108779 | O |
| O11 | 1.0 | 0.901088 | 0.108167 | 0.319443 | O |
| O12 | 1.0 | 0.421397 | 0.426836 | 0.999488 | O |
| O13 | 1.0 | 0.399752 | 0.422687 | 0.209564 | O |
| O14 | 1.0 | 0.349399 | 0.459011 | 0.423588 | O |
| O15 | 1.0 | 0.161162 | 0.915648 | 0.060206 | O |
| O16 | 1.0 | 0.155682 | 0.907326 | 0.267459 | O |
| O17 | 1.0 | 0.646804 | 0.582088 | 0.158380 | O |
| O18 | 1.0 | 0.638885 | 0.589620 | 0.379247 | O |
| O19 | 1.0 | 0.909879 | 0.405488 | 0.107293 | O |
| O20 | 1.0 | 0.906185 | 0.410968 | 0.319885 | O |
| O21 | 1.0 | 0.423676 | 0.087517 | 0.000850 | O |
| O22 | 1.0 | 0.399038 | 0.085276 | 0.213286 | O |
| O23 | 1.0 | 0.433242 | 0.151370 | 0.448863 | O |
| O24 | 1.0 | 0.158967 | 0.599496 | 0.058812 | O |
| O25 | 1.0 | 0.158247 | 0.598853 | 0.266641 | O |
| O26 | 1.0 | 0.646398 | 0.923337 | 0.159765 | O |
| O27 | 1.0 | 0.656598 | 0.900214 | 0.386540 | O |
| O28 | 1.0 | 0.115218 | 0.912400 | 0.158116 | O |
| O29 | 1.0 | 0.107945 | 0.917818 | 0.364187 | O |
| O30 | 1.0 | 0.619791 | 0.605317 | 0.047068 | O |
| O31 | 1.0 | 0.621889 | 0.589424 | 0.259167 | O |
| O32 | 1.0 | 0.848004 | 0.119304 | 0.998647 | O |
| O33 | 1.0 | 0.865736 | 0.093410 | 0.207568 | O |
| O34 | 1.0 | 0.860184 | 0.080717 | 0.423242 | O |
| O35 | 1.0 | 0.368541 | 0.410650 | 0.095187 | O |
| O36 | 1.0 | 0.371357 | 0.410520 | 0.309106 | O |
| O37 | 1.0 | 0.140856 | 0.254879 | 0.157817 | O |
| O38 | 1.0 | 0.141113 | 0.247560 | 0.369739 | O |
| O39 | 1.0 | 0.641399 | 0.260330 | 0.057827 | O |
| O40 | 1.0 | 0.647898 | 0.254125 | 0.262151 | O |
| O41 | 1.0 | 0.893263 | 0.754927 | 0.109062 | O |
| O42 | 1.0 | 0.886538 | 0.741288 | 0.323430 | O |
| O43 | 1.0 | 0.333227 | 0.758422 | 0.999492 | O |
| O44 | 1.0 | 0.368685 | 0.754016 | 0.212698 | O |

| | | | | | |
|---|---|---|---|---|---|
| O45 | 1.0 | 0.349534 | 0.776355 | 0.424455 | O |
| O46 | 1.0 | 0.902932 | 0.759458 | 0.999535 | O |
| O47 | 1.0 | 0.897402 | 0.753153 | 0.210552 | O |
| O48 | 1.0 | 0.964270 | 0.713645 | 0.425268 | O |
| O49 | 1.0 | 0.385732 | 0.755260 | 0.103694 | O |
| O50 | 1.0 | 0.389613 | 0.752501 | 0.315629 | O |
| O51 | 1.0 | 0.125258 | 0.257644 | 0.053872 | O |
| O52 | 1.0 | 0.116790 | 0.254067 | 0.266009 | O |
| O53 | 1.0 | 0.648053 | 0.252371 | 0.163919 | O |
| O54 | 1.0 | 0.624181 | 0.254044 | 0.374885 | O |
| O55 | 1.0 | 0.117701 | 0.298000 | 0.481721 | O |
| O56 | 1.0 | 0.644366 | 0.970071 | 0.499747 | O |
| Al1 | 1.0 | 0.396595 | 0.927902 | 0.055970 | Al |
| Al2 | 1.0 | 0.393809 | 0.925308 | 0.269246 | Al |
| Al3 | 1.0 | 0.140103 | 0.425627 | 0.111445 | Al |
| Al4 | 1.0 | 0.143285 | 0.412341 | 0.319894 | Al |
| Al5 | 1.0 | 0.640387 | 0.430767 | 0.013662 | Al |
| Al6 | 1.0 | 0.625883 | 0.428248 | 0.213944 | Al |
| Al7 | 1.0 | 0.600066 | 0.427930 | 0.423068 | Al |
| Al8 | 1.0 | 0.871843 | 0.923062 | 0.162576 | Al |
| Al9 | 1.0 | 0.900949 | 0.894152 | 0.391853 | Al |
| Al10 | 1.0 | 0.646109 | 0.081643 | 0.013449 | Al |
| Al11 | 1.0 | 0.626771 | 0.077917 | 0.214738 | Al |
| Al12 | 1.0 | 0.636791 | 0.039557 | 0.437749 | Al |
| Al13 | 1.0 | 0.871849 | 0.584469 | 0.162040 | Al |
| Al14 | 1.0 | 0.871069 | 0.572901 | 0.376016 | Al |
| Al15 | 1.0 | 0.394550 | 0.588439 | 0.054525 | Al |
| Al16 | 1.0 | 0.394471 | 0.581973 | 0.268389 | Al |
| Al17 | 1.0 | 0.144107 | 0.081770 | 0.113767 | Al |
| Al18 | 1.0 | 0.134109 | 0.080999 | 0.322633 | Al |
| Al19 | 1.0 | 0.898979 | 0.245267 | 0.053265 | Al |
| Al20 | 1.0 | 0.881748 | 0.258503 | 0.271649 | Al |
| Al21 | 1.0 | 0.509082 | 0.260270 | 0.116128 | Al |
| Al22 | 1.0 | 0.506438 | 0.248153 | 0.319780 | Al |
| Al23 | 1.0 | 0.886093 | 0.253226 | 0.161517 | Al |
| Al24 | 1.0 | 0.865835 | 0.239222 | 0.376911 | Al |
| Al25 | 1.0 | 0.105029 | 0.760190 | 0.014987 | Al |
| Al26 | 1.0 | 0.136600 | 0.753706 | 0.218841 | Al |
| Al27 | 1.0 | 0.198504 | 0.632965 | 0.423595 | Al |
| Al28 | 1.0 | 0.517792 | 0.752739 | 0.163589 | Al |
| Al29 | 1.0 | 0.501539 | 0.754008 | 0.379385 | Al |
| Al30 | 1.0 | 0.140113 | 0.754163 | 0.112582 | Al |
| Al31 | 1.0 | 0.129084 | 0.754056 | 0.321303 | Al |
| Al32 | 1.0 | 0.771397 | 0.758233 | 0.051973 | Al |
| Al33 | 1.0 | 0.755889 | 0.753181 | 0.270756 | Al |
| Al34 | 1.0 | 0.327714 | 0.257262 | 0.029553 | Al |
| Al35 | 1.0 | 0.270742 | 0.255243 | 0.218564 | Al |
| Al36 | 1.0 | 0.248800 | 0.273813 | 0.430931 | Al |

**Coordinate** for a γ-$Al_2O_3$ (100) surface with $CH_3$* and H* in Figure 4.
Spin Multiplicity <$S^2$>: 0.007
Absolute Energy: –719.813245 eV.

| | |
|---|---|
| cell_length_a | 8.07075 |
| cell_length_b | 8.40443 |
| cell_length_c | 26.38316 |
| cell_angle_alpha | 90 |
| cell_angle_beta | 90 |
| cell_angle_gamma | 90 |
| symmetry_space_group_name_H-M | 'P 1' |
| symmetry_Int_Tables_number | 1 |

| | | | | | |
|---|---|---|---|---|---|
| H1 | 1.0 | -0.004326 | 0.114684 | 0.471138 | H |
| H2 | 1.0 | 0.396313 | 0.760229 | 0.967201 | H |
| H3 | 1.0 | 0.340699 | 0.011573 | 0.438921 | H |
| H4 | 1.0 | 0.678107 | 0.493944 | 0.515064 | H |
| H5 | 1.0 | 0.461261 | 0.470217 | 0.501968 | H |
| H6 | 1.0 | 0.590864 | 0.299604 | 0.511193 | H |
| H7 | 1.0 | 0.875143 | 0.330013 | 0.970016 | H |
| H8 | 1.0 | 0.726134 | 0.985651 | 0.505465 | H |
| C1 | 1.0 | 0.584738 | 0.420596 | 0.495413 | C |
| O1 | 1.0 | 0.116963 | 0.595673 | 0.156889 | O |
| O2 | 1.0 | 0.127031 | 0.583898 | 0.361491 | O |
| O3 | 1.0 | 0.623370 | 0.909859 | 0.047825 | O |
| O4 | 1.0 | 0.625803 | 0.922371 | 0.260012 | O |
| O5 | 1.0 | 0.852515 | 0.415672 | 0.994563 | O |
| O6 | 1.0 | 0.868931 | 0.416026 | 0.207254 | O |
| O7 | 1.0 | 0.872482 | 0.411024 | 0.415214 | O |
| O8 | 1.0 | 0.374490 | 0.104932 | 0.096133 | O |
| O9 | 1.0 | 0.371849 | 0.091680 | 0.312306 | O |
| O10 | 1.0 | 0.916527 | 0.101232 | 0.108425 | O |
| O11 | 1.0 | 0.904701 | 0.110176 | 0.316610 | O |
| O12 | 1.0 | 0.424901 | 0.427211 | 0.999764 | O |
| O13 | 1.0 | 0.402877 | 0.426410 | 0.212065 | O |
| O14 | 1.0 | 0.229187 | 0.452750 | 0.450958 | O |
| O15 | 1.0 | 0.164972 | 0.916449 | 0.059688 | O |
| O16 | 1.0 | 0.160988 | 0.910541 | 0.266955 | O |
| O17 | 1.0 | 0.650472 | 0.585679 | 0.158917 | O |
| O18 | 1.0 | 0.641266 | 0.585107 | 0.379536 | O |
| O19 | 1.0 | 0.913705 | 0.408118 | 0.107082 | O |
| O20 | 1.0 | 0.907356 | 0.415113 | 0.318240 | O |
| O21 | 1.0 | 0.427888 | 0.087161 | 0.999961 | O |
| O22 | 1.0 | 0.402894 | 0.088393 | 0.211796 | O |
| O23 | 1.0 | 0.391491 | 0.112797 | 0.427314 | O |
| O24 | 1.0 | 0.163568 | 0.600402 | 0.058958 | O |
| O25 | 1.0 | 0.163165 | 0.601261 | 0.266954 | O |
| O26 | 1.0 | 0.650657 | 0.927176 | 0.159254 | O |
| O27 | 1.0 | 0.650978 | 0.901542 | 0.382163 | O |
| O28 | 1.0 | 0.118821 | 0.915450 | 0.157633 | O |
| O29 | 1.0 | 0.108897 | 0.922304 | 0.363544 | O |
| O30 | 1.0 | 0.624352 | 0.605315 | 0.047140 | O |
| O31 | 1.0 | 0.626151 | 0.593090 | 0.259677 | O |
| O32 | 1.0 | 0.853148 | 0.119133 | 0.998216 | O |
| O33 | 1.0 | 0.869034 | 0.097899 | 0.207070 | O |
| O34 | 1.0 | 0.857302 | 0.083186 | 0.418245 | O |
| O35 | 1.0 | 0.373406 | 0.412702 | 0.096046 | O |
| O36 | 1.0 | 0.376920 | 0.417662 | 0.313114 | O |
| O37 | 1.0 | 0.145244 | 0.257361 | 0.157382 | O |
| O38 | 1.0 | 0.132699 | 0.251776 | 0.368848 | O |
| O39 | 1.0 | 0.645768 | 0.260115 | 0.057344 | O |
| O40 | 1.0 | 0.650207 | 0.258155 | 0.262771 | O |
| O41 | 1.0 | 0.897041 | 0.756852 | 0.108850 | O |
| O42 | 1.0 | 0.888893 | 0.747190 | 0.322234 | O |
| O43 | 1.0 | 0.337813 | 0.758303 | 0.999452 | O |
| O44 | 1.0 | 0.372714 | 0.757188 | 0.212478 | O |

| | | | | | |
|---|---|---|---|---|---|
| O45 | 1.0 | 0.343279 | 0.758062 | 0.423731 | O |
| O46 | 1.0 | 0.907298 | 0.758639 | 0.999254 | O |
| O47 | 1.0 | 0.901589 | 0.756533 | 0.210252 | O |
| O48 | 1.0 | 0.949849 | 0.721497 | 0.422927 | O |
| O49 | 1.0 | 0.390133 | 0.757377 | 0.103506 | O |
| O50 | 1.0 | 0.391773 | 0.755765 | 0.315568 | O |
| O51 | 1.0 | 0.129501 | 0.259032 | 0.053685 | O |
| O52 | 1.0 | 0.122325 | 0.260175 | 0.265663 | O |
| O53 | 1.0 | 0.650225 | 0.257051 | 0.163843 | O |
| O54 | 1.0 | 0.627870 | 0.252732 | 0.378451 | O |
| O55 | 1.0 | 0.110388 | 0.136214 | 0.481041 | O |
| O56 | 1.0 | 0.622031 | 0.019165 | 0.490685 | O |
| Al1 | 1.0 | 0.400716 | 0.928118 | 0.055188 | Al |
| Al2 | 1.0 | 0.398005 | 0.928747 | 0.268527 | Al |
| Al3 | 1.0 | 0.144332 | 0.428055 | 0.111255 | Al |
| Al4 | 1.0 | 0.147228 | 0.417449 | 0.320148 | Al |
| Al5 | 1.0 | 0.644240 | 0.431057 | 0.013445 | Al |
| Al6 | 1.0 | 0.628937 | 0.432042 | 0.214289 | Al |
| Al7 | 1.0 | 0.653507 | 0.427700 | 0.425303 | Al |
| Al8 | 1.0 | 0.875919 | 0.926438 | 0.162111 | Al |
| Al9 | 1.0 | 0.892324 | 0.897468 | 0.387192 | Al |
| Al10 | 1.0 | 0.650870 | 0.081580 | 0.012481 | Al |
| Al11 | 1.0 | 0.629915 | 0.082712 | 0.214452 | Al |
| Al12 | 1.0 | 0.621760 | 0.065672 | 0.426422 | Al |
| Al13 | 1.0 | 0.875714 | 0.587463 | 0.161850 | Al |
| Al14 | 1.0 | 0.877923 | 0.578601 | 0.371540 | Al |
| Al15 | 1.0 | 0.399266 | 0.588814 | 0.054929 | Al |
| Al16 | 1.0 | 0.398834 | 0.583147 | 0.269081 | Al |
| Al17 | 1.0 | 0.147847 | 0.084120 | 0.113173 | Al |
| Al18 | 1.0 | 0.138861 | 0.084000 | 0.321493 | Al |
| Al19 | 1.0 | 0.903063 | 0.246714 | 0.052460 | Al |
| Al20 | 1.0 | 0.886181 | 0.262611 | 0.269675 | Al |
| Al21 | 1.0 | 0.513448 | 0.259784 | 0.115332 | Al |
| Al22 | 1.0 | 0.509749 | 0.252317 | 0.321821 | Al |
| Al23 | 1.0 | 0.888794 | 0.256622 | 0.160708 | Al |
| Al24 | 1.0 | 0.889578 | 0.242327 | 0.374261 | Al |
| Al25 | 1.0 | 0.109491 | 0.760092 | 0.014665 | Al |
| Al26 | 1.0 | 0.141156 | 0.755348 | 0.218921 | Al |
| Al27 | 1.0 | 0.168364 | 0.636762 | 0.428145 | Al |
| Al28 | 1.0 | 0.521579 | 0.756624 | 0.163369 | Al |
| Al29 | 1.0 | 0.500764 | 0.749241 | 0.380059 | Al |
| Al30 | 1.0 | 0.144067 | 0.756419 | 0.112403 | Al |
| Al31 | 1.0 | 0.140586 | 0.758313 | 0.321915 | Al |
| Al32 | 1.0 | 0.775638 | 0.758745 | 0.051649 | Al |
| Al33 | 1.0 | 0.758913 | 0.758292 | 0.269582 | Al |
| Al34 | 1.0 | 0.331225 | 0.256146 | 0.028750 | Al |
| Al35 | 1.0 | 0.274456 | 0.257429 | 0.217978 | Al |
| Al36 | 1.0 | 0.216650 | 0.253043 | 0.437741 | Al |

**Coordinate** for a calculated transition state of PT from $CH_3$* on a γ-$Al_2O_3$ (100) surface (**TS2**) in Figure 4.
Spin Multiplicity $<S^2>$: 0.9998
Absolute Energy: –718.93307207 eV.

| | |
|---|---|
| cell_length_a | 8.07075 |
| cell_length_b | 8.40443 |
| cell_length_c | 26.38316 |
| cell_angle_alpha | 90 |
| cell_angle_beta | 90 |
| cell_angle_gamma | 90 |
| symmetry_space_group_name_H-M | 'P 1' |
| symmetry_Int_Tables_number | 1 |

| | | | | | |
|---|---|---|---|---|---|
| H1 | 1.0 | 0.000663 | 0.098484 | 0.472939 | H |
| H2 | 1.0 | 0.396912 | 0.756187 | 0.967692 | H |
| H3 | 1.0 | 0.355404 | 0.042160 | 0.458310 | H |
| H4 | 1.0 | 0.569990 | 0.537756 | 0.513039 | H |
| H5 | 1.0 | 0.406994 | 0.617345 | 0.449918 | H |
| H6 | 1.0 | 0.485566 | 0.339893 | 0.503906 | H |
| H7 | 1.0 | 0.915912 | 0.381922 | 0.973496 | H |
| H8 | 1.0 | 0.729544 | 1.008597 | 0.508057 | H |
| C1 | 1.0 | 0.547045 | 0.441050 | 0.486035 | C |
| O1 | 1.0 | 0.120565 | 0.598794 | 0.158752 | O |
| O2 | 1.0 | 0.125383 | 0.581729 | 0.361714 | O |
| O3 | 1.0 | 0.628490 | 0.910692 | 0.045091 | O |
| O4 | 1.0 | 0.628314 | 0.922044 | 0.261775 | O |
| O5 | 1.0 | 0.874987 | 0.422266 | 0.005803 | O |
| O6 | 1.0 | 0.870680 | 0.413376 | 0.209338 | O |
| O7 | 1.0 | 0.872812 | 0.411366 | 0.414755 | O |
| O8 | 1.0 | 0.380443 | 0.104325 | 0.096707 | O |
| O9 | 1.0 | 0.375803 | 0.086628 | 0.314925 | O |
| O10 | 1.0 | 0.918223 | 0.100509 | 0.111144 | O |
| O11 | 1.0 | 0.910629 | 0.104167 | 0.318073 | O |
| O12 | 1.0 | 0.406694 | 0.425967 | 0.003096 | O |
| O13 | 1.0 | 0.405188 | 0.424012 | 0.215668 | O |
| O14 | 1.0 | 0.208745 | 0.448376 | 0.456177 | O |
| O15 | 1.0 | 0.168280 | 0.915592 | 0.060751 | O |
| O16 | 1.0 | 0.163321 | 0.907638 | 0.267751 | O |
| O17 | 1.0 | 0.652457 | 0.584320 | 0.160713 | O |
| O18 | 1.0 | 0.644802 | 0.582467 | 0.376281 | O |
| O19 | 1.0 | 0.918301 | 0.410038 | 0.111017 | O |
| O20 | 1.0 | 0.910240 | 0.408566 | 0.318695 | O |
| O21 | 1.0 | 0.424616 | 0.080839 | 0.999729 | O |
| O22 | 1.0 | 0.404279 | 0.085175 | 0.214548 | O |
| O23 | 1.0 | 0.394740 | 0.118196 | 0.432529 | O |
| O24 | 1.0 | 0.165748 | 0.600441 | 0.060988 | O |
| O25 | 1.0 | 0.164625 | 0.599654 | 0.267849 | O |
| O26 | 1.0 | 0.652603 | 0.925691 | 0.160927 | O |
| O27 | 1.0 | 0.646141 | 0.899344 | 0.384869 | O |
| O28 | 1.0 | 0.121897 | 0.913822 | 0.158363 | O |
| O29 | 1.0 | 0.117397 | 0.914956 | 0.365218 | O |
| O30 | 1.0 | 0.625061 | 0.598315 | 0.042565 | O |
| O31 | 1.0 | 0.629387 | 0.590772 | 0.261505 | O |
| O32 | 1.0 | 0.866567 | 0.111268 | 0.001625 | O |
| O33 | 1.0 | 0.870635 | 0.097596 | 0.209123 | O |
| O34 | 1.0 | 0.859022 | 0.081272 | 0.417596 | O |
| O35 | 1.0 | 0.379013 | 0.419829 | 0.101983 | O |
| O36 | 1.0 | 0.379486 | 0.418772 | 0.315762 | O |
| O37 | 1.0 | 0.148336 | 0.255353 | 0.159299 | O |
| O38 | 1.0 | 0.135903 | 0.249948 | 0.370361 | O |
| O39 | 1.0 | 0.651295 | 0.261489 | 0.056814 | O |
| O40 | 1.0 | 0.652681 | 0.255029 | 0.264359 | O |
| O41 | 1.0 | 0.895484 | 0.755261 | 0.108235 | O |
| O42 | 1.0 | 0.889032 | 0.749246 | 0.321978 | O |
| O43 | 1.0 | 0.338254 | 0.757043 | 0.999788 | O |
| O44 | 1.0 | 0.377545 | 0.754988 | 0.214036 | O |

| | | | | | |
|---|---|---|---|---|---|
| O45 | 1.0 | 0.360350 | 0.706795 | 0.428420 | O |
| O46 | 1.0 | 0.914182 | 0.756822 | 0.997505 | O |
| O47 | 1.0 | 0.903685 | 0.755934 | 0.210312 | O |
| O48 | 1.0 | 0.942266 | 0.720401 | 0.422287 | O |
| O49 | 1.0 | 0.395821 | 0.757383 | 0.103689 | O |
| O50 | 1.0 | 0.390590 | 0.753704 | 0.317220 | O |
| O51 | 1.0 | 0.132650 | 0.255642 | 0.058170 | O |
| O52 | 1.0 | 0.128094 | 0.255528 | 0.267359 | O |
| O53 | 1.0 | 0.648411 | 0.255405 | 0.164884 | O |
| O54 | 1.0 | 0.626325 | 0.250001 | 0.378802 | O |
| O55 | 1.0 | 0.115258 | 0.122495 | 0.481851 | O |
| O56 | 1.0 | 0.625826 | 0.038580 | 0.492180 | O |
| Al1 | 1.0 | 0.407330 | 0.927410 | 0.054332 | Al |
| Al2 | 1.0 | 0.400547 | 0.928424 | 0.267938 | Al |
| Al3 | 1.0 | 0.151125 | 0.427717 | 0.112534 | Al |
| Al4 | 1.0 | 0.150887 | 0.415117 | 0.319086 | Al |
| Al5 | 1.0 | 0.644007 | 0.422818 | 0.003990 | Al |
| Al6 | 1.0 | 0.632371 | 0.430670 | 0.214493 | Al |
| Al7 | 1.0 | 0.654230 | 0.425111 | 0.421487 | Al |
| Al8 | 1.0 | 0.878422 | 0.926549 | 0.160137 | Al |
| Al9 | 1.0 | 0.893716 | 0.897774 | 0.384698 | Al |
| Al10 | 1.0 | 0.656572 | 0.087745 | 0.008909 | Al |
| Al11 | 1.0 | 0.631617 | 0.080199 | 0.214026 | Al |
| Al12 | 1.0 | 0.624890 | 0.068868 | 0.426530 | Al |
| Al13 | 1.0 | 0.878168 | 0.584467 | 0.160145 | Al |
| Al14 | 1.0 | 0.876298 | 0.575699 | 0.367880 | Al |
| Al15 | 1.0 | 0.403561 | 0.581084 | 0.055172 | Al |
| Al16 | 1.0 | 0.401712 | 0.580068 | 0.268545 | Al |
| Al17 | 1.0 | 0.151911 | 0.085074 | 0.112396 | Al |
| Al18 | 1.0 | 0.145278 | 0.079746 | 0.322243 | Al |
| Al19 | 1.0 | 0.902135 | 0.249431 | 0.052301 | Al |
| Al20 | 1.0 | 0.888997 | 0.253901 | 0.268415 | Al |
| Al21 | 1.0 | 0.521372 | 0.262485 | 0.112660 | Al |
| Al22 | 1.0 | 0.511387 | 0.250749 | 0.320644 | Al |
| Al23 | 1.0 | 0.891545 | 0.255763 | 0.161640 | Al |
| Al24 | 1.0 | 0.895630 | 0.240816 | 0.373692 | Al |
| Al25 | 1.0 | 0.113617 | 0.758940 | 0.015237 | Al |
| Al26 | 1.0 | 0.143116 | 0.753350 | 0.217142 | Al |
| Al27 | 1.0 | 0.143115 | 0.622243 | 0.428438 | Al |
| Al28 | 1.0 | 0.522675 | 0.755080 | 0.162397 | Al |
| Al29 | 1.0 | 0.505705 | 0.744315 | 0.376432 | Al |
| Al30 | 1.0 | 0.147830 | 0.756266 | 0.112153 | Al |
| Al31 | 1.0 | 0.141062 | 0.755724 | 0.320408 | Al |
| Al32 | 1.0 | 0.775731 | 0.753500 | 0.048283 | Al |
| Al33 | 1.0 | 0.762506 | 0.757479 | 0.267077 | Al |
| Al34 | 1.0 | 0.324584 | 0.247461 | 0.026250 | Al |
| Al35 | 1.0 | 0.275808 | 0.254662 | 0.217260 | Al |
| Al36 | 1.0 | 0.209086 | 0.249486 | 0.438825 | Al |

**Coordinate** for a γ-$Al_2O_3$ (100) surface with $CH_2$* and 2H* in Figure 4.
Spin Multiplicity <$S^2$>: 1.000
Absolute Energy: –719.36810744 eV.

| | |
|---|---|
| cell_length_a | 8.07075 |
| cell_length_b | 8.40443 |
| cell_length_c | 26.38316 |
| cell_angle_alpha | 90 |
| cell_angle_beta | 90 |
| cell_angle_gamma | 90 |
| symmetry_space_group_name_H-M | 'P 1' |
| symmetry_Int_Tables_number | 1 |

| | | | | | |
|---|---|---|---|---|---|
| H1 | 1.0 | 0.079779 | 0.095029 | 0.474032 | H |
| H2 | 1.0 | 0.397238 | 0.758259 | 0.968677 | H |
| H3 | 1.0 | 0.357707 | 0.157557 | 0.472574 | H |
| H4 | 1.0 | 0.603288 | 0.320753 | 0.514956 | H |
| H5 | 1.0 | 0.427236 | 0.808728 | 0.454746 | H |
| H6 | 1.0 | 0.419664 | 0.409670 | 0.489593 | H |
| H7 | 1.0 | 0.916659 | 0.369523 | 0.973787 | H |
| H8 | 1.0 | 0.684742 | 0.826576 | 0.482903 | H |
| C1 | 1.0 | 0.531145 | 0.340427 | 0.480117 | C |
| O1 | 1.0 | 0.121353 | 0.600596 | 0.159491 | O |
| O2 | 1.0 | 0.123568 | 0.593266 | 0.363603 | O |
| O3 | 1.0 | 0.626054 | 0.915197 | 0.039483 | O |
| O4 | 1.0 | 0.627913 | 0.922997 | 0.262659 | O |
| O5 | 1.0 | 0.880715 | 0.415935 | 0.005880 | O |
| O6 | 1.0 | 0.870565 | 0.411351 | 0.211296 | O |
| O7 | 1.0 | 0.879607 | 0.409240 | 0.419945 | O |
| O8 | 1.0 | 0.384737 | 0.090903 | 0.103519 | O |
| O9 | 1.0 | 0.376251 | 0.087098 | 0.318723 | O |
| O10 | 1.0 | 0.920717 | 0.099535 | 0.113344 | O |
| O11 | 1.0 | 0.910493 | 0.099070 | 0.320589 | O |
| O12 | 1.0 | 0.395071 | 0.429703 | 0.001965 | O |
| O13 | 1.0 | 0.404050 | 0.422950 | 0.218525 | O |
| O14 | 1.0 | 0.178255 | 0.519761 | 0.462814 | O |
| O15 | 1.0 | 0.166705 | 0.914035 | 0.062202 | O |
| O16 | 1.0 | 0.163582 | 0.911087 | 0.268103 | O |
| O17 | 1.0 | 0.653450 | 0.586146 | 0.163009 | O |
| O18 | 1.0 | 0.646876 | 0.591286 | 0.383010 | O |
| O19 | 1.0 | 0.920165 | 0.411699 | 0.112884 | O |
| O20 | 1.0 | 0.903819 | 0.411520 | 0.321816 | O |
| O21 | 1.0 | 0.402242 | 0.079584 | 0.000606 | O |
| O22 | 1.0 | 0.403408 | 0.088154 | 0.218131 | O |
| O23 | 1.0 | 0.476024 | 0.181952 | 0.460761 | O |
| O24 | 1.0 | 0.165534 | 0.600134 | 0.061994 | O |
| O25 | 1.0 | 0.162087 | 0.600224 | 0.268844 | O |
| O26 | 1.0 | 0.653096 | 0.925128 | 0.162403 | O |
| O27 | 1.0 | 0.648280 | 0.916291 | 0.376687 | O |
| O28 | 1.0 | 0.122031 | 0.911006 | 0.159463 | O |
| O29 | 1.0 | 0.118320 | 0.917499 | 0.365632 | O |
| O30 | 1.0 | 0.625623 | 0.593553 | 0.039312 | O |
| O31 | 1.0 | 0.629333 | 0.587967 | 0.263685 | O |
| O32 | 1.0 | 0.879793 | 0.099433 | 0.004726 | O |
| O33 | 1.0 | 0.870219 | 0.099378 | 0.210961 | O |
| O34 | 1.0 | 0.859060 | 0.086542 | 0.420877 | O |
| O35 | 1.0 | 0.383189 | 0.423625 | 0.104382 | O |
| O36 | 1.0 | 0.377777 | 0.423218 | 0.318619 | O |
| O37 | 1.0 | 0.147189 | 0.255793 | 0.160798 | O |
| O38 | 1.0 | 0.124604 | 0.265577 | 0.373730 | O |
| O39 | 1.0 | 0.658713 | 0.259204 | 0.058293 | O |
| O40 | 1.0 | 0.653322 | 0.254561 | 0.265591 | O |
| O41 | 1.0 | 0.889930 | 0.755689 | 0.107561 | O |
| O42 | 1.0 | 0.886862 | 0.753173 | 0.320384 | O |
| O43 | 1.0 | 0.336950 | 0.756031 | 1.000531 | O |
| O44 | 1.0 | 0.378588 | 0.754679 | 0.214870 | O |

| | | | | | |
|---|---|---|---|---|---|
| O45 | 1.0 | 0.360321 | 0.770179 | 0.424507 | O |
| O46 | 1.0 | 0.917166 | 0.751242 | 0.995804 | O |
| O47 | 1.0 | 0.904146 | 0.755899 | 0.210164 | O |
| O48 | 1.0 | 0.929007 | 0.743496 | 0.419779 | O |
| O49 | 1.0 | 0.399276 | 0.756265 | 0.103390 | O |
| O50 | 1.0 | 0.389177 | 0.754057 | 0.316716 | O |
| O51 | 1.0 | 0.135567 | 0.255375 | 0.060405 | O |
| O52 | 1.0 | 0.127168 | 0.256458 | 0.270445 | O |
| O53 | 1.0 | 0.644221 | 0.255980 | 0.165449 | O |
| O54 | 1.0 | 0.637476 | 0.255373 | 0.375665 | O |
| O55 | 1.0 | 0.153410 | 0.185340 | 0.479809 | O |
| O56 | 1.0 | 0.596360 | 0.904684 | 0.479278 | O |
| Al1 | 1.0 | 0.408178 | 0.934694 | 0.053845 | Al |
| Al2 | 1.0 | 0.400732 | 0.930643 | 0.267648 | Al |
| Al3 | 1.0 | 0.155411 | 0.427125 | 0.111603 | Al |
| Al4 | 1.0 | 0.148791 | 0.423542 | 0.319581 | Al |
| Al5 | 1.0 | 0.643209 | 0.409991 | 0.000243 | Al |
| Al6 | 1.0 | 0.632123 | 0.430459 | 0.213680 | Al |
| Al7 | 1.0 | 0.651572 | 0.419574 | 0.420742 | Al |
| Al8 | 1.0 | 0.878671 | 0.925491 | 0.157790 | Al |
| Al9 | 1.0 | 0.887641 | 0.915107 | 0.375289 | Al |
| Al10 | 1.0 | 0.661471 | 0.097459 | 0.000751 | Al |
| Al11 | 1.0 | 0.631218 | 0.080496 | 0.213066 | Al |
| Al12 | 1.0 | 0.639194 | 0.047698 | 0.430433 | Al |
| Al13 | 1.0 | 0.878810 | 0.585425 | 0.157592 | Al |
| Al14 | 1.0 | 0.878338 | 0.588647 | 0.368872 | Al |
| Al15 | 1.0 | 0.405746 | 0.578288 | 0.054253 | Al |
| Al16 | 1.0 | 0.401535 | 0.580929 | 0.267802 | Al |
| Al17 | 1.0 | 0.157388 | 0.084697 | 0.112202 | Al |
| Al18 | 1.0 | 0.149922 | 0.087425 | 0.323640 | Al |
| Al19 | 1.0 | 0.898938 | 0.244944 | 0.052523 | Al |
| Al20 | 1.0 | 0.887232 | 0.253727 | 0.266480 | Al |
| Al21 | 1.0 | 0.519482 | 0.257911 | 0.110234 | Al |
| Al22 | 1.0 | 0.510186 | 0.253916 | 0.319183 | Al |
| Al23 | 1.0 | 0.891926 | 0.256169 | 0.161806 | Al |
| Al24 | 1.0 | 0.878552 | 0.243499 | 0.370784 | Al |
| Al25 | 1.0 | 0.114313 | 0.759396 | 0.015223 | Al |
| Al26 | 1.0 | 0.142446 | 0.755189 | 0.214890 | Al |
| Al27 | 1.0 | 0.147274 | 0.686564 | 0.425673 | Al |
| Al28 | 1.0 | 0.521598 | 0.755444 | 0.161020 | Al |
| Al29 | 1.0 | 0.510662 | 0.755810 | 0.372960 | Al |
| Al30 | 1.0 | 0.152382 | 0.755995 | 0.111672 | Al |
| Al31 | 1.0 | 0.140528 | 0.760338 | 0.318041 | Al |
| Al32 | 1.0 | 0.770914 | 0.755334 | 0.044189 | Al |
| Al33 | 1.0 | 0.760781 | 0.756533 | 0.263898 | Al |
| Al34 | 1.0 | 0.295973 | 0.251953 | 0.013661 | Al |
| Al35 | 1.0 | 0.272525 | 0.255636 | 0.217071 | Al |
| Al36 | 1.0 | 0.098190 | 0.345192 | 0.439948 | Al |

## S3.2 Optimized Structures and Energies for Figure S6

**Coordinate** for a γ-$Al_2O_3$ (100) surface with $CH_4$ σ-complex at a 5-coordinated Al site.
Absolute Energy: –731.75658299 eV.

| | |
|---|---|
| cell_length_a | 8.070750 |
| cell_length_b | 8.404430 |
| cell_length_c | 26.383160 |
| cell_angle_alpha | 90.000000 |
| cell_angle_beta | 90.000000 |
| cell_angle_gamma | 90.000000 |
| cell_volume | 1789.571263 |
| space_group_name_H-M_alt | 'P 1' |
| space_group_IT_number | 1 |

| | | | | | |
|---|---|---|---|---|---|
| H1 | 1.0 | 0.917929 | 0.402544 | 0.444165 | H |
| H2 | 1.0 | 0.430293 | 0.072259 | 0.963119 | H |
| H3 | 1.0 | 0.698900 | 0.423558 | 0.558535 | H |
| H4 | 1.0 | 0.730352 | 0.582663 | 0.514720 | H |
| H5 | 1.0 | 0.525923 | 0.532422 | 0.535311 | H |
| H6 | 1.0 | 0.666923 | 0.627423 | 0.578229 | H |
| H7 | 1.0 | 0.122325 | 0.404754 | 0.500383 | H |
| H8 | 1.0 | 0.381135 | 0.753554 | 0.962556 | H |
| H9 | 1.0 | 0.828333 | 0.320928 | 0.952131 | H |
| H10 | 1.0 | 0.683783 | 0.834203 | 0.495196 | H |
| C1 | 1.0 | 0.655790 | 0.541785 | 0.547048 | C |
| O1 | 1.0 | 0.604802 | 0.267201 | 0.465222 | O |
| O2 | 1.0 | 0.109730 | 0.590496 | 0.154556 | O |
| O3 | 1.0 | 0.118468 | 0.569548 | 0.362387 | O |
| O4 | 1.0 | 0.623466 | 0.914940 | 0.035307 | O |
| O5 | 1.0 | 0.615635 | 0.912945 | 0.257862 | O |
| O6 | 1.0 | 0.768406 | 0.421576 | 0.956204 | O |
| O7 | 1.0 | 0.857361 | 0.407709 | 0.206065 | O |
| O8 | 1.0 | 0.844424 | 0.403586 | 0.414270 | O |
| O9 | 1.0 | 0.374276 | 0.112646 | 0.087387 | O |
| O10 | 1.0 | 0.358715 | 0.080144 | 0.311606 | O |
| O11 | 1.0 | 0.904955 | 0.088489 | 0.108383 | O |
| O12 | 1.0 | 0.896492 | 0.099626 | 0.314347 | O |
| O13 | 1.0 | 0.396195 | 0.414241 | -0.002523 | O |
| O14 | 1.0 | 0.390985 | 0.419350 | 0.208524 | O |
| O15 | 1.0 | 0.372114 | 0.429061 | 0.412812 | O |
| O16 | 1.0 | 0.159240 | 0.915713 | 0.058801 | O |
| O17 | 1.0 | 0.150357 | 0.899657 | 0.266537 | O |
| O18 | 1.0 | 0.639829 | 0.575840 | 0.154991 | O |
| O19 | 1.0 | 0.635591 | 0.585039 | 0.377484 | O |
| O20 | 1.0 | 0.903535 | 0.402527 | 0.108172 | O |
| O21 | 1.0 | 0.899693 | 0.403782 | 0.318544 | O |
| O22 | 1.0 | 0.354199 | 0.072712 | -0.007826 | O |
| O23 | 1.0 | 0.390596 | 0.077464 | 0.210980 | O |
| O24 | 1.0 | 0.394823 | 0.104127 | 0.438144 | O |
| O25 | 1.0 | 0.146444 | 0.602103 | 0.055203 | O |
| O26 | 1.0 | 0.153712 | 0.593196 | 0.266492 | O |
| O27 | 1.0 | 0.639858 | 0.915177 | 0.156130 | O |
| O28 | 1.0 | 0.652038 | 0.905160 | 0.391172 | O |
| O29 | 1.0 | 0.111136 | 0.908170 | 0.155449 | O |
| O30 | 1.0 | 0.095962 | 0.913099 | 0.364212 | O |
| O31 | 1.0 | 0.608673 | 0.597327 | 0.037566 | O |
| O32 | 1.0 | 0.615430 | 0.583986 | 0.256325 | O |
| O33 | 1.0 | 0.882552 | 0.131634 | -0.004844 | O |
| O34 | 1.0 | 0.856913 | 0.086509 | 0.205219 | O |
| O35 | 1.0 | 0.864681 | 0.089454 | 0.423095 | O |
| O36 | 1.0 | 0.361784 | 0.415886 | 0.094312 | O |
| O37 | 1.0 | 0.363630 | 0.403622 | 0.307228 | O |
| O38 | 1.0 | 0.136280 | 0.246876 | 0.156200 | O |
| O39 | 1.0 | 0.125258 | 0.242344 | 0.367612 | O |

| | | | | | |
|---|---|---|---|---|---|
| O40 | 1.0 | 0.653850 | 0.276956 | 0.052860 | O |
| O41 | 1.0 | 0.639738 | 0.247904 | 0.257526 | O |
| O42 | 1.0 | 0.884933 | 0.748002 | 0.106051 | O |
| O43 | 1.0 | 0.884514 | 0.729793 | 0.317959 | O |
| O44 | 1.0 | 0.322869 | 0.751616 | -0.005031 | O |
| O45 | 1.0 | 0.361172 | 0.748195 | 0.210311 | O |
| O46 | 1.0 | 0.347875 | 0.743636 | 0.426894 | O |
| O47 | 1.0 | 0.890003 | 0.788142 | -0.001557 | O |
| O48 | 1.0 | 0.891849 | 0.746854 | 0.207378 | O |
| O49 | 1.0 | 0.933763 | 0.707033 | 0.423979 | O |
| O50 | 1.0 | 0.387970 | 0.755409 | 0.098207 | O |
| O51 | 1.0 | 0.385257 | 0.748502 | 0.315627 | O |
| O52 | 1.0 | 0.109821 | 0.258259 | 0.056550 | O |
| O53 | 1.0 | 0.109794 | 0.250978 | 0.263153 | O |
| O54 | 1.0 | 0.638194 | 0.246269 | 0.159947 | O |
| O55 | 1.0 | 0.635417 | 0.252517 | 0.363681 | O |
| O56 | 1.0 | 0.123261 | 0.405014 | 0.463552 | O |
| O57 | 1.0 | 0.654476 | 0.945339 | 0.499832 | O |
| Al1 | 1.0 | 0.404301 | 0.930189 | 0.048975 | Al |
| Al2 | 1.0 | 0.387492 | 0.918819 | 0.267365 | Al |
| Al3 | 1.0 | 0.134695 | 0.419214 | 0.110633 | Al |
| Al4 | 1.0 | 0.135111 | 0.409157 | 0.316515 | Al |
| Al5 | 1.0 | 0.620626 | 0.417023 | 0.002354 | Al |
| Al6 | 1.0 | 0.616051 | 0.422591 | 0.210667 | Al |
| Al7 | 1.0 | 0.592059 | 0.402196 | 0.412065 | Al |
| Al8 | 1.0 | 0.866548 | 0.917168 | 0.159876 | Al |
| Al9 | 1.0 | 0.892460 | 0.895831 | 0.397868 | Al |
| Al10 | 1.0 | 0.759752 | -0.029019 | -0.019659 | Al |
| Al11 | 1.0 | 0.617312 | 0.071677 | 0.210614 | Al |
| Al12 | 1.0 | 0.624204 | 0.059495 | 0.443816 | Al |
| Al13 | 1.0 | 0.866364 | 0.576373 | 0.159069 | Al |
| Al14 | 1.0 | 0.863482 | 0.581133 | 0.371262 | Al |
| Al15 | 1.0 | 0.383929 | 0.581840 | 0.049298 | Al |
| Al16 | 1.0 | 0.389975 | 0.576565 | 0.267520 | Al |
| Al17 | 1.0 | 0.141190 | 0.078655 | 0.110946 | Al |
| Al18 | 1.0 | 0.125043 | 0.071328 | 0.319852 | Al |
| Al19 | 1.0 | 0.878344 | 0.225629 | 0.054976 | Al |
| Al20 | 1.0 | 0.874268 | 0.254232 | 0.268168 | Al |
| Al21 | 1.0 | 0.519161 | 0.270225 | 0.107086 | Al |
| Al22 | 1.0 | 0.499311 | 0.240240 | 0.314002 | Al |
| Al23 | 1.0 | 0.878954 | 0.247401 | 0.160352 | Al |
| Al24 | 1.0 | 0.865546 | 0.227228 | 0.370896 | Al |
| Al25 | 1.0 | 0.099310 | 0.767719 | 0.013284 | Al |
| Al26 | 1.0 | 0.129852 | 0.746273 | 0.217808 | Al |
| Al27 | 1.0 | 0.213769 | 0.586978 | 0.423923 | Al |
| Al28 | 1.0 | 0.509477 | 0.745884 | 0.160709 | Al |
| Al29 | 1.0 | 0.501723 | 0.755516 | 0.381262 | Al |
| Al30 | 1.0 | 0.140656 | 0.751019 | 0.110238 | Al |
| Al31 | 1.0 | 0.123964 | 0.748670 | 0.322216 | Al |
| Al32 | 1.0 | 0.750431 | 0.748250 | 0.052716 | Al |
| Al33 | 1.0 | 0.749996 | 0.748173 | 0.267106 | Al |
| Al34 | 1.0 | 0.304434 | 0.248574 | 0.027808 | Al |
| Al35 | 1.0 | 0.264936 | 0.249360 | 0.215914 | Al |
| Al36 | 1.0 | 0.256962 | 0.254941 | 0.423897 | Al |

**Coordinate** for a calculated transition state of the initial $CH_4$ activation on a γ-$Al_2O_3$ (100) surface (**TS1**) in Figure S6.
Absolute Energy: –731.15875929 eV.

| | |
|---|---|
| cell_length_a | 8.070750 |
| cell_length_b | 8.404430 |
| cell_length_c | 26.383160 |
| cell_angle_alpha | 90.000000 |
| cell_angle_beta | 90.000000 |
| cell_angle_gamma | 90.000000 |
| cell_volume | 1789.571263 |
| space_group_name_H-M_alt | 'P 1' |
| space_group_IT_number | 1 |

| | | | | | |
|---|---|---|---|---|---|
| H1 | 1.0 | 0.920240 | 0.400297 | 0.445216 | H |
| H2 | 1.0 | 0.446076 | 0.056069 | 0.966456 | H |
| H3 | 1.0 | 0.561919 | 0.045875 | 0.520166 | H |
| H4 | 1.0 | 0.755645 | 0.658765 | 0.491000 | H |
| H5 | 1.0 | 0.521510 | 0.642216 | 0.495072 | H |
| H6 | 1.0 | 0.654002 | 0.584338 | 0.550936 | H |
| H7 | 1.0 | 0.127609 | 0.406266 | 0.501422 | H |
| H8 | 1.0 | 0.392015 | 0.762486 | 0.967252 | H |
| H9 | 1.0 | 0.821895 | 0.302536 | 0.953363 | H |
| H10 | 1.0 | 0.613689 | 0.868908 | 0.504787 | H |
| C1 | 1.0 | 0.643463 | 0.633358 | 0.512911 | C |
| O1 | 1.0 | 0.610577 | 0.276015 | 0.468214 | O |
| O2 | 1.0 | 0.113988 | 0.596167 | 0.158018 | O |
| O3 | 1.0 | 0.118020 | 0.571217 | 0.362424 | O |
| O4 | 1.0 | 0.626232 | 0.911975 | 0.039562 | O |
| O5 | 1.0 | 0.618653 | 0.917782 | 0.260709 | O |
| O6 | 1.0 | 0.772178 | 0.407821 | 0.958558 | O |
| O7 | 1.0 | 0.859057 | 0.408682 | 0.209112 | O |
| O8 | 1.0 | 0.846876 | 0.404233 | 0.415123 | O |
| O9 | 1.0 | 0.373672 | 0.114030 | 0.088357 | O |
| O10 | 1.0 | 0.361968 | 0.081373 | 0.314132 | O |
| O11 | 1.0 | 0.907286 | 0.090389 | 0.110455 | O |
| O12 | 1.0 | 0.899208 | 0.102416 | 0.314982 | O |
| O13 | 1.0 | 0.398394 | 0.408191 | 0.001087 | O |
| O14 | 1.0 | 0.393592 | 0.423396 | 0.212612 | O |
| O15 | 1.0 | 0.370005 | 0.441124 | 0.416136 | O |
| O16 | 1.0 | 0.159311 | 0.915029 | 0.061107 | O |
| O17 | 1.0 | 0.152807 | 0.901854 | 0.267916 | O |
| O18 | 1.0 | 0.642895 | 0.580726 | 0.159124 | O |
| O19 | 1.0 | 0.638874 | 0.587873 | 0.380216 | O |
| O20 | 1.0 | 0.907042 | 0.411381 | 0.112283 | O |
| O21 | 1.0 | 0.898203 | 0.404932 | 0.319865 | O |
| O22 | 1.0 | 0.355829 | 0.063164 | 0.992858 | O |
| O23 | 1.0 | 0.392555 | 0.080863 | 0.213547 | O |
| O24 | 1.0 | 0.391845 | 0.111184 | 0.438487 | O |
| O25 | 1.0 | 0.149614 | 0.601396 | 0.058658 | O |
| O26 | 1.0 | 0.154973 | 0.595946 | 0.268258 | O |
| O27 | 1.0 | 0.641762 | 0.919997 | 0.159435 | O |
| O28 | 1.0 | 0.650073 | 0.900787 | 0.393952 | O |
| O29 | 1.0 | 0.114311 | 0.910955 | 0.157936 | O |
| O30 | 1.0 | 0.100448 | 0.915917 | 0.364996 | O |
| O31 | 1.0 | 0.612412 | 0.592096 | 0.040479 | O |
| O32 | 1.0 | 0.619561 | 0.585933 | 0.260068 | O |
| O33 | 1.0 | 0.884407 | 0.109602 | 0.994427 | O |
| O34 | 1.0 | 0.859028 | 0.093033 | 0.206950 | O |
| O35 | 1.0 | 0.862193 | 0.087925 | 0.421755 | O |
| O36 | 1.0 | 0.365508 | 0.419917 | 0.098834 | O |
| O37 | 1.0 | 0.367960 | 0.407995 | 0.310923 | O |
| O38 | 1.0 | 0.139875 | 0.249967 | 0.157440 | O |
| O39 | 1.0 | 0.125166 | 0.246149 | 0.368439 | O |
| O40 | 1.0 | 0.661563 | 0.272238 | 0.054814 | O |
| O41 | 1.0 | 0.641205 | 0.250435 | 0.260303 | O |
| O42 | 1.0 | 0.885766 | 0.751125 | 0.108905 | O |

| | | | | | |
|---|---|---|---|---|---|
| O43 | 1.0 | 0.882053 | 0.738707 | 0.321977 | O |
| O44 | 1.0 | 0.327203 | 0.746235 | 0.998405 | O |
| O45 | 1.0 | 0.367114 | 0.751074 | 0.213673 | O |
| O46 | 1.0 | 0.344930 | 0.747810 | 0.429936 | O |
| O47 | 1.0 | 0.893103 | 0.774880 | 0.999668 | O |
| O48 | 1.0 | 0.894479 | 0.751045 | 0.210349 | O |
| O49 | 1.0 | 0.949767 | 0.706879 | 0.425416 | O |
| O50 | 1.0 | 0.389301 | 0.756959 | 0.101557 | O |
| O51 | 1.0 | 0.385043 | 0.750348 | 0.318190 | O |
| O52 | 1.0 | 0.107422 | 0.258430 | 0.057218 | O |
| O53 | 1.0 | 0.114563 | 0.253782 | 0.264832 | O |
| O54 | 1.0 | 0.636685 | 0.251026 | 0.161815 | O |
| O55 | 1.0 | 0.630414 | 0.243521 | 0.369417 | O |
| O56 | 1.0 | 0.117073 | 0.407649 | 0.464748 | O |
| O57 | 1.0 | 0.640459 | 0.983044 | 0.500351 | O |
| Al1 | 1.0 | 0.408163 | 0.927698 | 0.050890 | Al |
| Al2 | 1.0 | 0.390853 | 0.921653 | 0.268445 | Al |
| Al3 | 1.0 | 0.136249 | 0.423987 | 0.111240 | Al |
| Al4 | 1.0 | 0.138404 | 0.412362 | 0.316438 | Al |
| Al5 | 1.0 | 0.623802 | 0.412822 | 0.004500 | Al |
| Al6 | 1.0 | 0.619066 | 0.426970 | 0.212547 | Al |
| Al7 | 1.0 | 0.589616 | 0.410655 | 0.415413 | Al |
| Al8 | 1.0 | 0.868619 | 0.920402 | 0.161249 | Al |
| Al9 | 1.0 | 0.894893 | 0.893784 | 0.395571 | Al |
| Al10 | 1.0 | 0.742888 | 0.961881 | 0.975011 | Al |
| Al11 | 1.0 | 0.619449 | 0.075289 | 0.212131 | Al |
| Al12 | 1.0 | 0.622716 | 0.084181 | 0.432368 | Al |
| Al13 | 1.0 | 0.869795 | 0.579683 | 0.160712 | Al |
| Al14 | 1.0 | 0.866937 | 0.579032 | 0.371240 | Al |
| Al15 | 1.0 | 0.387853 | 0.579404 | 0.051866 | Al |
| Al16 | 1.0 | 0.393587 | 0.577947 | 0.268850 | Al |
| Al17 | 1.0 | 0.144303 | 0.080666 | 0.112388 | Al |
| Al18 | 1.0 | 0.128708 | 0.073601 | 0.319764 | Al |
| Al19 | 1.0 | 0.878989 | 0.205986 | 0.052359 | Al |
| Al20 | 1.0 | 0.876852 | 0.255260 | 0.267273 | Al |
| Al21 | 1.0 | 0.520766 | 0.269963 | 0.107407 | Al |
| Al22 | 1.0 | 0.501603 | 0.242285 | 0.315711 | Al |
| Al23 | 1.0 | 0.878590 | 0.253156 | 0.160588 | Al |
| Al24 | 1.0 | 0.873496 | 0.231605 | 0.370736 | Al |
| Al25 | 1.0 | 0.101318 | 0.765943 | 0.014800 | Al |
| Al26 | 1.0 | 0.133436 | 0.748095 | 0.217851 | Al |
| Al27 | 1.0 | 0.193733 | 0.603274 | 0.425551 | Al |
| Al28 | 1.0 | 0.511702 | 0.749897 | 0.162104 | Al |
| Al29 | 1.0 | 0.496121 | 0.753721 | 0.382436 | Al |
| Al30 | 1.0 | 0.142457 | 0.753596 | 0.111988 | Al |
| Al31 | 1.0 | 0.128607 | 0.751714 | 0.322341 | Al |
| Al32 | 1.0 | 0.755689 | 0.746584 | 0.053224 | Al |
| Al33 | 1.0 | 0.752683 | 0.752398 | 0.268505 | Al |
| Al34 | 1.0 | 0.298903 | 0.238997 | 0.025993 | Al |
| Al35 | 1.0 | 0.266690 | 0.252792 | 0.216001 | Al |
| Al36 | 1.0 | 0.257031 | 0.265115 | 0.423848 | Al |

**Coordinate** for a γ-$Al_2O_3$ (100) surface with $CH_3$* and H* in Figure S6.
Absolute Energy: –735.48943316 eV.

| | |
|---|---|
| cell_length_a | 8.070750 |
| cell_length_b | 8.404430 |
| cell_length_c | 26.383160 |
| cell_angle_alpha | 90.000000 |
| cell_angle_beta | 90.000000 |
| cell_angle_gamma | 90.000000 |
| cell_volume | 1789.571263 |
| space_group_name_H-M_alt | 'P 1' |
| space_group_IT_number | 1 |

| | | | | | |
|---|---|---|---|---|---|
| H1 | 1.0 | 0.920806 | 0.416708 | 0.445179 | H |
| H2 | 1.0 | 0.454286 | 0.063327 | 0.966430 | H |
| H3 | 1.0 | 0.319484 | 0.002360 | 0.433871 | H |
| H4 | 1.0 | 0.610200 | 0.432055 | 0.533295 | H |
| H5 | 1.0 | 0.429203 | 0.317138 | 0.519836 | H |
| H6 | 1.0 | 0.607844 | 0.220170 | 0.544821 | H |
| H7 | 1.0 | 0.131170 | 0.431022 | 0.500998 | H |
| H8 | 1.0 | 0.403265 | 0.775152 | 0.968250 | H |
| H9 | 1.0 | 0.823690 | 0.312047 | 0.951249 | H |
| H10 | 1.0 | 0.565867 | 0.880729 | 0.500626 | H |
| C1 | 1.0 | 0.566060 | 0.315602 | 0.519540 | C |
| O1 | 1.0 | 0.629214 | 0.287386 | 0.469707 | O |
| O2 | 1.0 | 0.120958 | 0.609141 | 0.158642 | O |
| O3 | 1.0 | 0.123781 | 0.583731 | 0.362801 | O |
| O4 | 1.0 | 0.633554 | 0.921791 | 0.040463 | O |
| O5 | 1.0 | 0.624030 | 0.930817 | 0.261153 | O |
| O6 | 1.0 | 0.775984 | 0.417061 | 0.958030 | O |
| O7 | 1.0 | 0.864648 | 0.420422 | 0.209722 | O |
| O8 | 1.0 | 0.850064 | 0.417351 | 0.414479 | O |
| O9 | 1.0 | 0.379645 | 0.125096 | 0.088326 | O |
| O10 | 1.0 | 0.365763 | 0.093327 | 0.314335 | O |
| O11 | 1.0 | 0.913268 | 0.102080 | 0.110653 | O |
| O12 | 1.0 | 0.902982 | 0.114064 | 0.315228 | O |
| O13 | 1.0 | 0.404731 | 0.417564 | 0.001913 | O |
| O14 | 1.0 | 0.398809 | 0.435425 | 0.213792 | O |
| O15 | 1.0 | 0.383596 | 0.446087 | 0.420575 | O |
| O16 | 1.0 | 0.165675 | 0.925754 | 0.061224 | O |
| O17 | 1.0 | 0.157698 | 0.913849 | 0.268013 | O |
| O18 | 1.0 | 0.648939 | 0.593536 | 0.160096 | O |
| O19 | 1.0 | 0.642544 | 0.599670 | 0.380230 | O |
| O20 | 1.0 | 0.913921 | 0.425834 | 0.113374 | O |
| O21 | 1.0 | 0.901764 | 0.417868 | 0.319645 | O |
| O22 | 1.0 | 0.363145 | 0.072478 | 0.992842 | O |
| O23 | 1.0 | 0.397786 | 0.092710 | 0.213814 | O |
| O24 | 1.0 | 0.367009 | 0.110529 | 0.437763 | O |
| O25 | 1.0 | 0.156671 | 0.612322 | 0.059022 | O |
| O26 | 1.0 | 0.159789 | 0.608008 | 0.268607 | O |
| O27 | 1.0 | 0.647699 | 0.932317 | 0.160068 | O |
| O28 | 1.0 | 0.652105 | 0.914421 | 0.392026 | O |
| O29 | 1.0 | 0.120046 | 0.923033 | 0.158196 | O |
| O30 | 1.0 | 0.105374 | 0.928505 | 0.364923 | O |
| O31 | 1.0 | 0.619621 | 0.601400 | 0.040918 | O |
| O32 | 1.0 | 0.625433 | 0.598184 | 0.260976 | O |
| O33 | 1.0 | 0.890484 | 0.114251 | 0.993335 | O |
| O34 | 1.0 | 0.864824 | 0.106006 | 0.207138 | O |
| O35 | 1.0 | 0.868617 | 0.097643 | 0.422011 | O |
| O36 | 1.0 | 0.372015 | 0.431685 | 0.099719 | O |
| O37 | 1.0 | 0.372490 | 0.420723 | 0.312229 | O |
| O38 | 1.0 | 0.146178 | 0.261689 | 0.157514 | O |
| O39 | 1.0 | 0.126302 | 0.260623 | 0.368269 | O |
| O40 | 1.0 | 0.670228 | 0.281948 | 0.054988 | O |
| O41 | 1.0 | 0.645782 | 0.262523 | 0.260853 | O |
| O42 | 1.0 | 0.891963 | 0.762955 | 0.109344 | O |

| | | | | | |
|---|---|---|---|---|---|
| O43 | 1.0 | 0.885777 | 0.753487 | 0.322731 | O |
| O44 | 1.0 | 0.335771 | 0.756578 | 0.998919 | O |
| O45 | 1.0 | 0.373262 | 0.763733 | 0.214218 | O |
| O46 | 1.0 | 0.341864 | 0.767765 | 0.428459 | O |
| O47 | 1.0 | 0.901048 | 0.781583 | -0.000281 | O |
| O48 | 1.0 | 0.900288 | 0.763616 | 0.210822 | O |
| O49 | 1.0 | 0.954323 | 0.721193 | 0.424718 | O |
| O50 | 1.0 | 0.395053 | 0.768540 | 0.102068 | O |
| O51 | 1.0 | 0.389108 | 0.762285 | 0.318387 | O |
| O52 | 1.0 | 0.111845 | 0.268335 | 0.057096 | O |
| O53 | 1.0 | 0.119944 | 0.265090 | 0.265232 | O |
| O54 | 1.0 | 0.641680 | 0.263530 | 0.162095 | O |
| O55 | 1.0 | 0.633988 | 0.255726 | 0.370235 | O |
| O56 | 1.0 | 0.115711 | 0.433238 | 0.464435 | O |
| O57 | 1.0 | 0.638776 | 0.972081 | 0.499111 | O |
| Al1 | 1.0 | 0.415591 | 0.938032 | 0.051018 | Al |
| Al2 | 1.0 | 0.396086 | 0.934220 | 0.268303 | Al |
| Al3 | 1.0 | 0.142218 | 0.436055 | 0.111207 | Al |
| Al4 | 1.0 | 0.142748 | 0.425175 | 0.316518 | Al |
| Al5 | 1.0 | 0.630292 | 0.422385 | 0.004623 | Al |
| Al6 | 1.0 | 0.624646 | 0.439721 | 0.212925 | Al |
| Al7 | 1.0 | 0.598674 | 0.419666 | 0.414565 | Al |
| Al8 | 1.0 | 0.874628 | 0.932628 | 0.161255 | Al |
| Al9 | 1.0 | 0.894226 | 0.905432 | 0.392905 | Al |
| Al10 | 1.0 | 0.744590 | 0.970201 | 0.973157 | Al |
| Al11 | 1.0 | 0.624848 | 0.087798 | 0.212247 | Al |
| Al12 | 1.0 | 0.640832 | 0.075552 | 0.441410 | Al |
| Al13 | 1.0 | 0.876165 | 0.592058 | 0.160951 | Al |
| Al14 | 1.0 | 0.869910 | 0.589373 | 0.369804 | Al |
| Al15 | 1.0 | 0.395006 | 0.590083 | 0.052282 | Al |
| Al16 | 1.0 | 0.399207 | 0.589429 | 0.269270 | Al |
| Al17 | 1.0 | 0.150830 | 0.092438 | 0.112353 | Al |
| Al18 | 1.0 | 0.132979 | 0.086488 | 0.319347 | Al |
| Al19 | 1.0 | 0.884415 | 0.209426 | 0.051011 | Al |
| Al20 | 1.0 | 0.881350 | 0.266949 | 0.266954 | Al |
| Al21 | 1.0 | 0.526935 | 0.280774 | 0.107071 | Al |
| Al22 | 1.0 | 0.505964 | 0.253918 | 0.316323 | Al |
| Al23 | 1.0 | 0.883750 | 0.265950 | 0.160372 | Al |
| Al24 | 1.0 | 0.873677 | 0.241921 | 0.370820 | Al |
| Al25 | 1.0 | 0.109101 | 0.776530 | 0.014627 | Al |
| Al26 | 1.0 | 0.139485 | 0.759947 | 0.217543 | Al |
| Al27 | 1.0 | 0.175511 | 0.635521 | 0.426826 | Al |
| Al28 | 1.0 | 0.517217 | 0.762452 | 0.162268 | Al |
| Al29 | 1.0 | 0.500324 | 0.764139 | 0.382083 | Al |
| Al30 | 1.0 | 0.148203 | 0.765356 | 0.112013 | Al |
| Al31 | 1.0 | 0.136194 | 0.764030 | 0.322135 | Al |
| Al32 | 1.0 | 0.763408 | 0.756533 | 0.052927 | Al |
| Al33 | 1.0 | 0.758065 | 0.765458 | 0.268358 | Al |
| Al34 | 1.0 | 0.303104 | 0.247893 | 0.025366 | Al |
| Al35 | 1.0 | 0.271816 | 0.264537 | 0.215925 | Al |
| Al36 | 1.0 | 0.252243 | 0.284856 | 0.423719 | Al |

**Coordinate** for a calculated transition state from $CH_3$* on a γ-$Al_2O_3$ (100) surface (**TS3**) in Figure S6.
Absolute Energy: –734.90592846 eV.

| | |
|---|---|
| cell_length_a | 8.070750 |
| cell_length_b | 8.404430 |
| cell_length_c | 26.383160 |
| cell_angle_alpha | 90.000000 |
| cell_angle_beta | 90.000000 |
| cell_angle_gamma | 90.000000 |
| cell_volume | 1789.571263 |
| space_group_name_H-M_alt | 'P 1' |
| space_group_IT_number | 1 |

| | | | | | |
|---|---|---|---|---|---|
| H1 | 1.0 | 0.878798 | 0.413373 | 0.449579 | H |
| H2 | 1.0 | 0.452120 | 0.055973 | 0.966720 | H |
| H3 | 1.0 | 0.316144 | 0.998889 | 0.431882 | H |
| H4 | 1.0 | 0.535542 | 0.543100 | 0.533514 | H |
| H5 | 1.0 | 0.479016 | 0.335993 | 0.529587 | H |
| H6 | 1.0 | 0.667816 | 0.392185 | 0.559001 | H |
| H7 | 1.0 | 0.174586 | 0.444668 | 0.498809 | H |
| H8 | 1.0 | 0.399931 | 0.766877 | 0.968159 | H |
| H9 | 1.0 | 0.824437 | 0.303567 | 0.952502 | H |
| H10 | 1.0 | 0.529432 | 1.014650 | 0.509586 | H |
| C1 | 1.0 | 0.583355 | 0.421278 | 0.527514 | C |
| O1 | 1.0 | 0.669836 | 0.409237 | 0.481470 | O |
| O2 | 1.0 | 0.118922 | 0.601528 | 0.158589 | O |
| O3 | 1.0 | 0.122411 | 0.578516 | 0.362271 | O |
| O4 | 1.0 | 0.631296 | 0.914454 | 0.040661 | O |
| O5 | 1.0 | 0.622810 | 0.923657 | 0.261260 | O |
| O6 | 1.0 | 0.777559 | 0.409065 | 0.959267 | O |
| O7 | 1.0 | 0.862922 | 0.412872 | 0.210034 | O |
| O8 | 1.0 | 0.860943 | 0.410207 | 0.412601 | O |
| O9 | 1.0 | 0.377788 | 0.117161 | 0.088756 | O |
| O10 | 1.0 | 0.367387 | 0.086839 | 0.314780 | O |
| O11 | 1.0 | 0.911337 | 0.094688 | 0.111065 | O |
| O12 | 1.0 | 0.902623 | 0.106943 | 0.315619 | O |
| O13 | 1.0 | 0.403408 | 0.409843 | 0.001996 | O |
| O14 | 1.0 | 0.398040 | 0.428328 | 0.213399 | O |
| O15 | 1.0 | 0.391747 | 0.433740 | 0.417281 | O |
| O16 | 1.0 | 0.163287 | 0.918036 | 0.061343 | O |
| O17 | 1.0 | 0.157471 | 0.908251 | 0.267418 | O |
| O18 | 1.0 | 0.647402 | 0.585604 | 0.160008 | O |
| O19 | 1.0 | 0.644326 | 0.592268 | 0.376634 | O |
| O20 | 1.0 | 0.912119 | 0.417707 | 0.113552 | O |
| O21 | 1.0 | 0.900452 | 0.410379 | 0.319534 | O |
| O22 | 1.0 | 0.360912 | 0.064984 | 0.993171 | O |
| O23 | 1.0 | 0.396753 | 0.085903 | 0.214060 | O |
| O24 | 1.0 | 0.365080 | 0.106808 | 0.435061 | O |
| O25 | 1.0 | 0.154560 | 0.604239 | 0.059083 | O |
| O26 | 1.0 | 0.158087 | 0.600886 | 0.267971 | O |
| O27 | 1.0 | 0.645607 | 0.925268 | 0.160234 | O |
| O28 | 1.0 | 0.647451 | 0.901646 | 0.387172 | O |
| O29 | 1.0 | 0.118332 | 0.915209 | 0.158248 | O |
| O30 | 1.0 | 0.107641 | 0.921669 | 0.364073 | O |
| O31 | 1.0 | 0.617721 | 0.593891 | 0.041129 | O |
| O32 | 1.0 | 0.624239 | 0.591678 | 0.260658 | O |
| O33 | 1.0 | 0.888785 | 0.106469 | 0.993923 | O |
| O34 | 1.0 | 0.862968 | 0.098772 | 0.207624 | O |
| O35 | 1.0 | 0.865528 | 0.085909 | 0.419529 | O |
| O36 | 1.0 | 0.370056 | 0.423646 | 0.099749 | O |
| O37 | 1.0 | 0.372204 | 0.414393 | 0.311614 | O |
| O38 | 1.0 | 0.144365 | 0.254120 | 0.157895 | O |
| O39 | 1.0 | 0.126692 | 0.254223 | 0.367873 | O |
| O40 | 1.0 | 0.667962 | 0.274343 | 0.055555 | O |
| O41 | 1.0 | 0.644325 | 0.255806 | 0.262739 | O |
| O42 | 1.0 | 0.889977 | 0.755460 | 0.109315 | O |

| | | | | | |
|---|---|---|---|---|---|
| O43 | 1.0 | 0.887045 | 0.749362 | 0.321828 | O |
| O44 | 1.0 | 0.333178 | 0.748619 | 0.998977 | O |
| O45 | 1.0 | 0.371977 | 0.756203 | 0.213879 | O |
| O46 | 1.0 | 0.338046 | 0.758886 | 0.426954 | O |
| O47 | 1.0 | 0.898593 | 0.773749 | 0.999710 | O |
| O48 | 1.0 | 0.898611 | 0.756461 | 0.210598 | O |
| O49 | 1.0 | 0.947432 | 0.718360 | 0.422907 | O |
| O50 | 1.0 | 0.393158 | 0.760583 | 0.102026 | O |
| O51 | 1.0 | 0.386234 | 0.754234 | 0.317219 | O |
| O52 | 1.0 | 0.110337 | 0.260407 | 0.057483 | O |
| O53 | 1.0 | 0.119167 | 0.258005 | 0.265454 | O |
| O54 | 1.0 | 0.639892 | 0.256083 | 0.162776 | O |
| O55 | 1.0 | 0.629883 | 0.242264 | 0.376539 | O |
| O56 | 1.0 | 0.127497 | 0.439986 | 0.464820 | O |
| O57 | 1.0 | 0.635859 | 0.035232 | 0.494013 | O |
| Al1 | 1.0 | 0.413379 | 0.930428 | 0.051153 | Al |
| Al2 | 1.0 | 0.395453 | 0.928106 | 0.268203 | Al |
| Al3 | 1.0 | 0.140422 | 0.428367 | 0.111402 | Al |
| Al4 | 1.0 | 0.142912 | 0.419303 | 0.316301 | Al |
| Al5 | 1.0 | 0.629038 | 0.414635 | 0.005042 | Al |
| Al6 | 1.0 | 0.623784 | 0.431925 | 0.213308 | Al |
| Al7 | 1.0 | 0.605383 | 0.429000 | 0.418312 | Al |
| Al8 | 1.0 | 0.872275 | 0.925268 | 0.161205 | Al |
| Al9 | 1.0 | 0.892563 | 0.898062 | 0.388390 | Al |
| Al10 | 1.0 | 0.742245 | 0.963489 | 0.973322 | Al |
| Al11 | 1.0 | 0.623494 | 0.080991 | 0.212824 | Al |
| Al12 | 1.0 | 0.633099 | 0.069319 | 0.429184 | Al |
| Al13 | 1.0 | 0.874292 | 0.584561 | 0.160929 | Al |
| Al14 | 1.0 | 0.870586 | 0.582597 | 0.368560 | Al |
| Al15 | 1.0 | 0.393106 | 0.582370 | 0.052360 | Al |
| Al16 | 1.0 | 0.397648 | 0.582274 | 0.268294 | Al |
| Al17 | 1.0 | 0.149073 | 0.084927 | 0.112575 | Al |
| Al18 | 1.0 | 0.134687 | 0.080473 | 0.318993 | Al |
| Al19 | 1.0 | 0.882531 | 0.202754 | 0.051482 | Al |
| Al20 | 1.0 | 0.879885 | 0.259388 | 0.267254 | Al |
| Al21 | 1.0 | 0.525055 | 0.272929 | 0.107670 | Al |
| Al22 | 1.0 | 0.505789 | 0.249104 | 0.319478 | Al |
| Al23 | 1.0 | 0.882676 | 0.258400 | 0.160787 | Al |
| Al24 | 1.0 | 0.882262 | 0.236028 | 0.371443 | Al |
| Al25 | 1.0 | 0.106593 | 0.768630 | 0.014641 | Al |
| Al26 | 1.0 | 0.138258 | 0.753806 | 0.217100 | Al |
| Al27 | 1.0 | 0.163192 | 0.636842 | 0.426819 | Al |
| Al28 | 1.0 | 0.515900 | 0.754681 | 0.162062 | Al |
| Al29 | 1.0 | 0.495151 | 0.750105 | 0.380452 | Al |
| Al30 | 1.0 | 0.146080 | 0.757648 | 0.111915 | Al |
| Al31 | 1.0 | 0.137664 | 0.757421 | 0.321260 | Al |
| Al32 | 1.0 | 0.761280 | 0.749215 | 0.052937 | Al |
| Al33 | 1.0 | 0.756599 | 0.758637 | 0.267966 | Al |
| Al34 | 1.0 | 0.301726 | 0.240482 | 0.025774 | Al |
| Al35 | 1.0 | 0.271195 | 0.257790 | 0.216217 | Al |
| Al36 | 1.0 | 0.251512 | 0.285227 | 0.423495 | Al |

**Coordinate** for a γ-$Al_2O_3$ (100) surface with $CH_3OH$* in Figure S6.
Absolute Energy: –735.56165834eV.

| | |
|---|---|
| cell_length_a | 8.070750 |
| cell_length_b | 8.404430 |
| cell_length_c | 26.383160 |
| cell_angle_alpha | 90.000000 |
| cell_angle_beta | 90.000000 |
| cell_angle_gamma | 90.000000 |
| cell_volume | 1789.571263 |
| space_group_name_H-M_alt | 'P 1' |
| space_group_IT_number | 1 |

| | | | | | |
|---|---|---|---|---|---|
| H1 | 1.0 | 0.878798 | 0.413373 | 0.449579 | H |
| H2 | 1.0 | 0.452120 | 0.055973 | 0.966720 | H |
| H3 | 1.0 | 0.316144 | 0.998889 | 0.431882 | H |
| H4 | 1.0 | 0.535542 | 0.543100 | 0.533514 | H |
| H5 | 1.0 | 0.479016 | 0.335993 | 0.529587 | H |
| H6 | 1.0 | 0.667816 | 0.392185 | 0.559001 | H |
| H7 | 1.0 | 0.174586 | 0.444668 | 0.498809 | H |
| H8 | 1.0 | 0.399931 | 0.766877 | 0.968159 | H |
| H9 | 1.0 | 0.824437 | 0.303567 | 0.952502 | H |
| H10 | 1.0 | 0.529432 | 1.014650 | 0.509586 | H |
| C1 | 1.0 | 0.583355 | 0.421278 | 0.527514 | C |
| O1 | 1.0 | 0.669836 | 0.409237 | 0.481470 | O |
| O2 | 1.0 | 0.118922 | 0.601528 | 0.158589 | O |
| O3 | 1.0 | 0.122411 | 0.578516 | 0.362271 | O |
| O4 | 1.0 | 0.631296 | 0.914454 | 0.040661 | O |
| O5 | 1.0 | 0.622810 | 0.923657 | 0.261260 | O |
| O6 | 1.0 | 0.777559 | 0.409065 | 0.959267 | O |
| O7 | 1.0 | 0.862922 | 0.412872 | 0.210034 | O |
| O8 | 1.0 | 0.860943 | 0.410207 | 0.412601 | O |
| O9 | 1.0 | 0.377788 | 0.117161 | 0.088756 | O |
| O10 | 1.0 | 0.367387 | 0.086839 | 0.314780 | O |
| O11 | 1.0 | 0.911337 | 0.094688 | 0.111065 | O |
| O12 | 1.0 | 0.902623 | 0.106943 | 0.315619 | O |
| O13 | 1.0 | 0.403408 | 0.409843 | 0.001996 | O |
| O14 | 1.0 | 0.398040 | 0.428328 | 0.213399 | O |
| O15 | 1.0 | 0.391747 | 0.433740 | 0.417281 | O |
| O16 | 1.0 | 0.163287 | 0.918036 | 0.061343 | O |
| O17 | 1.0 | 0.157471 | 0.908251 | 0.267418 | O |
| O18 | 1.0 | 0.647402 | 0.585604 | 0.160008 | O |
| O19 | 1.0 | 0.644326 | 0.592268 | 0.376634 | O |
| O20 | 1.0 | 0.912119 | 0.417707 | 0.113552 | O |
| O21 | 1.0 | 0.900452 | 0.410379 | 0.319534 | O |
| O22 | 1.0 | 0.360912 | 0.064984 | 0.993171 | O |
| O23 | 1.0 | 0.396753 | 0.085903 | 0.214060 | O |
| O24 | 1.0 | 0.365080 | 0.106808 | 0.435061 | O |
| O25 | 1.0 | 0.154560 | 0.604239 | 0.059083 | O |
| O26 | 1.0 | 0.158087 | 0.600886 | 0.267971 | O |
| O27 | 1.0 | 0.645607 | 0.925268 | 0.160234 | O |
| O28 | 1.0 | 0.647451 | 0.901646 | 0.387172 | O |
| O29 | 1.0 | 0.118332 | 0.915209 | 0.158248 | O |
| O30 | 1.0 | 0.107641 | 0.921669 | 0.364073 | O |
| O31 | 1.0 | 0.617721 | 0.593891 | 0.041129 | O |
| O32 | 1.0 | 0.624239 | 0.591678 | 0.260658 | O |
| O33 | 1.0 | 0.888785 | 0.106469 | 0.993923 | O |
| O34 | 1.0 | 0.862968 | 0.098772 | 0.207624 | O |
| O35 | 1.0 | 0.865528 | 0.085909 | 0.419529 | O |
| O36 | 1.0 | 0.370056 | 0.423646 | 0.099749 | O |
| O37 | 1.0 | 0.372204 | 0.414393 | 0.311614 | O |
| O38 | 1.0 | 0.144365 | 0.254120 | 0.157895 | O |
| O39 | 1.0 | 0.126692 | 0.254223 | 0.367873 | O |
| O40 | 1.0 | 0.667962 | 0.274343 | 0.055555 | O |
| O41 | 1.0 | 0.644325 | 0.255806 | 0.262739 | O |
| O42 | 1.0 | 0.889977 | 0.755460 | 0.109315 | O |

| | | | | | |
|---|---|---|---|---|---|
| O43 | 1.0 | 0.887045 | 0.749362 | 0.321828 | O |
| O44 | 1.0 | 0.333178 | 0.748619 | 0.998977 | O |
| O45 | 1.0 | 0.371977 | 0.756203 | 0.213879 | O |
| O46 | 1.0 | 0.338046 | 0.758886 | 0.426954 | O |
| O47 | 1.0 | 0.898593 | 0.773749 | 0.999710 | O |
| O48 | 1.0 | 0.898611 | 0.756461 | 0.210598 | O |
| O49 | 1.0 | 0.947432 | 0.718360 | 0.422907 | O |
| O50 | 1.0 | 0.393158 | 0.760583 | 0.102026 | O |
| O51 | 1.0 | 0.386234 | 0.754234 | 0.317219 | O |
| O52 | 1.0 | 0.110337 | 0.260407 | 0.057483 | O |
| O53 | 1.0 | 0.119167 | 0.258005 | 0.265454 | O |
| O54 | 1.0 | 0.639892 | 0.256083 | 0.162776 | O |
| O55 | 1.0 | 0.629883 | 0.242264 | 0.376539 | O |
| O56 | 1.0 | 0.127497 | 0.439986 | 0.464820 | O |
| O57 | 1.0 | 0.635859 | 0.035232 | 0.494013 | O |
| Al1 | 1.0 | 0.413379 | 0.930428 | 0.051153 | Al |
| Al2 | 1.0 | 0.395453 | 0.928106 | 0.268203 | Al |
| Al3 | 1.0 | 0.140422 | 0.428367 | 0.111402 | Al |
| Al4 | 1.0 | 0.142912 | 0.419303 | 0.316301 | Al |
| Al5 | 1.0 | 0.629038 | 0.414635 | 0.005042 | Al |
| Al6 | 1.0 | 0.623784 | 0.431925 | 0.213308 | Al |
| Al7 | 1.0 | 0.605383 | 0.429000 | 0.418312 | Al |
| Al8 | 1.0 | 0.872275 | 0.925268 | 0.161205 | Al |
| Al9 | 1.0 | 0.892563 | 0.898062 | 0.388390 | Al |
| Al10 | 1.0 | 0.742245 | 0.963489 | 0.973322 | Al |
| Al11 | 1.0 | 0.623494 | 0.080991 | 0.212824 | Al |
| Al12 | 1.0 | 0.633099 | 0.069319 | 0.429184 | Al |
| Al13 | 1.0 | 0.874292 | 0.584561 | 0.160929 | Al |
| Al14 | 1.0 | 0.870586 | 0.582597 | 0.368560 | Al |
| Al15 | 1.0 | 0.393106 | 0.582370 | 0.052360 | Al |
| Al16 | 1.0 | 0.397648 | 0.582274 | 0.268294 | Al |
| Al17 | 1.0 | 0.149073 | 0.084927 | 0.112575 | Al |
| Al18 | 1.0 | 0.134687 | 0.080473 | 0.318993 | Al |
| Al19 | 1.0 | 0.882531 | 0.202754 | 0.051482 | Al |
| Al20 | 1.0 | 0.879885 | 0.259388 | 0.267254 | Al |
| Al21 | 1.0 | 0.525055 | 0.272929 | 0.107670 | Al |
| Al22 | 1.0 | 0.505789 | 0.249104 | 0.319478 | Al |
| Al23 | 1.0 | 0.882676 | 0.258400 | 0.160787 | Al |
| Al24 | 1.0 | 0.882262 | 0.236028 | 0.371443 | Al |
| Al25 | 1.0 | 0.106593 | 0.768630 | 0.014641 | Al |
| Al26 | 1.0 | 0.138258 | 0.753806 | 0.217100 | Al |
| Al27 | 1.0 | 0.163192 | 0.636842 | 0.426819 | Al |
| Al28 | 1.0 | 0.515900 | 0.754681 | 0.162062 | Al |
| Al29 | 1.0 | 0.495151 | 0.750105 | 0.380452 | Al |
| Al30 | 1.0 | 0.146080 | 0.757648 | 0.111915 | Al |
| Al31 | 1.0 | 0.137664 | 0.757421 | 0.321260 | Al |
| Al32 | 1.0 | 0.761280 | 0.749215 | 0.052937 | Al |
| Al33 | 1.0 | 0.756599 | 0.758637 | 0.267966 | Al |
| Al34 | 1.0 | 0.301726 | 0.240482 | 0.025774 | Al |
| Al35 | 1.0 | 0.271195 | 0.257790 | 0.216217 | Al |
| Al36 | 1.0 | 0.251512 | 0.285227 | 0.423495 | Al |

## S3.3 Optimized Structures and Energies for Figure S8

**Coordinate** for a γ-$Al_2O_3$ (100) surface with $CH_4$ σ-complex at a 6-coordinated Al site.
Absolute Energy: –751.20248905 eV.

| | |
|---|---|
| cell_length_a | 8.070750 |
| cell_length_b | 8.404430 |
| cell_length_c | 26.383160 |
| cell_angle_alpha | 90.000000 |
| cell_angle_beta | 90.000000 |
| cell_angle_gamma | 90.000000 |
| cell_volume | 1789.571263 |
| space_group_name_H-M_alt | 'P 1' |
| space_group_IT_number | 1 |

| | | | | | |
|---|---|---|---|---|---|
| H1 | 1.0 | 0.000647 | 0.431891 | 0.446116 | H |
| H2 | 1.0 | 0.397608 | 0.758756 | 0.963031 | H |
| H3 | 1.0 | 0.870488 | 0.312798 | 0.966527 | H |
| H4 | 1.0 | 0.573832 | 0.796423 | 0.505100 | H |
| H5 | 1.0 | 0.272796 | 0.835634 | 0.398556 | H |
| H6 | 1.0 | 0.274106 | 0.002458 | 0.462014 | H |
| H7 | 1.0 | 0.843184 | 0.145696 | 0.447270 | H |
| H8 | 1.0 | 0.848942 | 0.716110 | 0.448037 | H |
| H9 | 1.0 | 0.658037 | 0.556409 | 0.550341 | H |
| H10 | 1.0 | 0.575993 | 0.516356 | 0.612460 | H |
| H11 | 1.0 | 0.454986 | 0.477515 | 0.556797 | H |
| H12 | 1.0 | 0.630540 | 0.355652 | 0.570290 | H |
| C1 | 1.0 | 0.580270 | 0.476538 | 0.572878 | C |
| O1 | 1.0 | 0.658774 | 0.227307 | 0.468866 | O |
| O2 | 1.0 | 0.620666 | 0.599222 | 0.455137 | O |
| O3 | 1.0 | 0.118417 | 0.581923 | 0.150846 | O |
| O4 | 1.0 | 0.127608 | 0.583215 | 0.361494 | O |
| O5 | 1.0 | 0.624612 | 0.903348 | 0.044615 | O |
| O6 | 1.0 | 0.621451 | 0.918417 | 0.263303 | O |
| O7 | 1.0 | 0.849375 | 0.407438 | -0.012199 | O |
| O8 | 1.0 | 0.868542 | 0.407173 | 0.203658 | O |
| O9 | 1.0 | 0.858931 | 0.430101 | 0.411832 | O |
| O10 | 1.0 | 0.376508 | 0.097723 | 0.093119 | O |
| O11 | 1.0 | 0.373472 | 0.092954 | 0.312919 | O |
| O12 | 1.0 | 0.914556 | 0.091267 | 0.105388 | O |
| O13 | 1.0 | 0.905570 | 0.102053 | 0.320048 | O |
| O14 | 1.0 | 0.426961 | 0.420271 | 0.994823 | O |
| O15 | 1.0 | 0.399640 | 0.417798 | 0.205912 | O |
| O16 | 1.0 | 0.397411 | 0.404877 | 0.415910 | O |
| O17 | 1.0 | 0.166605 | 0.910026 | 0.055735 | O |
| O18 | 1.0 | 0.155206 | 0.917205 | 0.264850 | O |
| O19 | 1.0 | 0.651776 | 0.575618 | 0.154193 | O |
| O20 | 1.0 | 0.657389 | 0.643572 | 0.354562 | O |
| O21 | 1.0 | 0.912459 | 0.395732 | 0.102188 | O |
| O22 | 1.0 | 0.909678 | 0.413748 | 0.314137 | O |
| O23 | 1.0 | 0.429336 | 0.081886 | 0.996628 | O |
| O24 | 1.0 | 0.402379 | 0.081175 | 0.211801 | O |
| O25 | 1.0 | 0.336166 | 0.092580 | 0.448538 | O |
| O26 | 1.0 | 0.164857 | 0.592937 | 0.053537 | O |
| O27 | 1.0 | 0.150858 | 0.599089 | 0.263520 | O |
| O28 | 1.0 | 0.649046 | 0.917548 | 0.159010 | O |
| O29 | 1.0 | 0.660241 | 0.932165 | 0.405259 | O |
| O30 | 1.0 | 0.124011 | 0.910491 | 0.153193 | O |
| O31 | 1.0 | 0.128808 | 0.921816 | 0.364706 | O |
| O32 | 1.0 | 0.625024 | 0.599693 | 0.042269 | O |
| O33 | 1.0 | 0.616713 | 0.587665 | 0.253960 | O |
| O34 | 1.0 | 0.852600 | 0.112314 | 0.994526 | O |
| O35 | 1.0 | 0.868774 | 0.091329 | 0.205271 | O |
| O36 | 1.0 | 0.929182 | 0.113219 | 0.421156 | O |
| O37 | 1.0 | 0.372846 | 0.402912 | 0.090047 | O |

| | | | | | |
|---|---|---|---|---|---|
| O38 | 1.0 | 0.369439 | 0.417061 | 0.305235 | O |
| O39 | 1.0 | 0.144400 | 0.248091 | 0.153272 | O |
| O40 | 1.0 | 0.137443 | 0.259692 | 0.362830 | O |
| O41 | 1.0 | 0.646442 | 0.254074 | 0.053410 | O |
| O42 | 1.0 | 0.651521 | 0.251663 | 0.258874 | O |
| O43 | 1.0 | 0.899196 | 0.750876 | 0.103411 | O |
| O44 | 1.0 | 0.902983 | 0.765765 | 0.320709 | O |
| O45 | 1.0 | 0.338669 | 0.753537 | 0.995096 | O |
| O46 | 1.0 | 0.363729 | 0.749509 | 0.207348 | O |
| O47 | 1.0 | 0.344124 | 0.756078 | 0.418599 | O |
| O48 | 1.0 | 0.908621 | 0.753784 | 0.994218 | O |
| O49 | 1.0 | 0.912066 | 0.750947 | 0.202258 | O |
| O50 | 1.0 | 0.925551 | 0.743724 | 0.420244 | O |
| O51 | 1.0 | 0.391618 | 0.748743 | 0.099374 | O |
| O52 | 1.0 | 0.378565 | 0.753182 | 0.307377 | O |
| O53 | 1.0 | 0.129561 | 0.248455 | 0.049615 | O |
| O54 | 1.0 | 0.117719 | 0.250326 | 0.262123 | O |
| O55 | 1.0 | 0.648983 | 0.246209 | 0.160587 | O |
| O56 | 1.0 | 0.647111 | 0.272644 | 0.366905 | O |
| O57 | 1.0 | 0.126317 | 0.426998 | 0.456738 | O |
| O58 | 1.0 | 0.541827 | 0.908124 | 0.506370 | O |
| Al1 | 1.0 | 0.401861 | 0.921285 | 0.052225 | Al |
| Al2 | 1.0 | 0.393965 | 0.930145 | 0.267270 | Al |
| Al3 | 1.0 | 0.142309 | 0.418910 | 0.106183 | Al |
| Al4 | 1.0 | 0.143593 | 0.422899 | 0.311547 | Al |
| Al5 | 1.0 | 0.645422 | 0.424806 | 0.009250 | Al |
| Al6 | 1.0 | 0.627990 | 0.425741 | 0.209596 | Al |
| Al7 | 1.0 | 0.628495 | 0.398498 | 0.430223 | Al |
| Al8 | 1.0 | 0.875979 | 0.916140 | 0.159125 | Al |
| Al9 | 1.0 | 0.897715 | 0.929024 | 0.382263 | Al |
| Al10 | 1.0 | 0.650718 | 0.075065 | 0.009500 | Al |
| Al11 | 1.0 | 0.630716 | 0.068353 | 0.213099 | Al |
| Al12 | 1.0 | 0.567775 | 0.045835 | 0.458439 | Al |
| Al13 | 1.0 | 0.878867 | 0.583495 | 0.160017 | Al |
| Al14 | 1.0 | 0.880217 | 0.590776 | 0.366740 | Al |
| Al15 | 1.0 | 0.399561 | 0.582899 | 0.050109 | Al |
| Al16 | 1.0 | 0.389785 | 0.583874 | 0.261335 | Al |
| Al17 | 1.0 | 0.147760 | 0.075825 | 0.108720 | Al |
| Al18 | 1.0 | 0.144175 | 0.084508 | 0.317994 | Al |
| Al19 | 1.0 | 0.902547 | 0.237668 | 0.049419 | Al |
| Al20 | 1.0 | 0.883336 | 0.249930 | 0.265298 | Al |
| Al21 | 1.0 | 0.514640 | 0.254474 | 0.111582 | Al |
| Al22 | 1.0 | 0.510793 | 0.259101 | 0.316501 | Al |
| Al23 | 1.0 | 0.886213 | 0.248304 | 0.156924 | Al |
| Al24 | 1.0 | 0.874555 | 0.261556 | 0.368724 | Al |
| Al25 | 1.0 | 0.110109 | 0.754742 | 0.010838 | Al |
| Al26 | 1.0 | 0.132613 | 0.761471 | 0.215760 | Al |
| Al27 | 1.0 | 0.251242 | 0.563824 | 0.418507 | Al |
| Al28 | 1.0 | 0.521506 | 0.745543 | 0.160587 | Al |
| Al29 | 1.0 | 0.571231 | 0.737221 | 0.408888 | Al |
| Al30 | 1.0 | 0.144454 | 0.747515 | 0.108852 | Al |
| Al31 | 1.0 | 0.144293 | 0.759536 | 0.317597 | Al |
| Al32 | 1.0 | 0.777551 | 0.751864 | 0.046971 | Al |
| Al33 | 1.0 | 0.694025 | 0.740907 | 0.293842 | Al |
| Al34 | 1.0 | 0.333444 | 0.251690 | 0.025708 | Al |
| Al35 | 1.0 | 0.271245 | 0.249691 | 0.213155 | Al |
| Al36 | 1.0 | 0.233931 | 0.263123 | 0.423719 | Al |

**Coordinate** for a calculated transition state of the $CH_4$ activation on a γ-$Al_2O_3$ (100) surface (**TS1**) in Figure S8.
Absolute Energy: –748.39713689 eV.

| | |
|---|---|
| cell_length_a | 8.070750 |
| cell_length_b | 8.404430 |
| cell_length_c | 26.383160 |
| cell_angle_alpha | 90.000000 |
| cell_angle_beta | 90.000000 |
| cell_angle_gamma | 90.000000 |
| cell_volume | 1789.571263 |
| space_group_name_H-M_alt | 'P 1' |
| space_group_IT_number | 1 |

| | | | | | |
|---|---|---|---|---|---|
| H1 | 1.0 | 0.026189 | 0.433421 | 0.450212 | H |
| H2 | 1.0 | 0.398855 | 0.757379 | 0.963082 | H |
| H3 | 1.0 | 0.894729 | 0.344723 | 0.967341 | H |
| H4 | 1.0 | 0.598421 | 0.894456 | 0.534915 | H |
| H5 | 1.0 | 0.257766 | 0.897019 | 0.417964 | H |
| H6 | 1.0 | 0.274648 | 0.065829 | 0.486932 | H |
| H7 | 1.0 | 0.954007 | 0.082717 | 0.447181 | H |
| H8 | 1.0 | 0.812876 | 0.702044 | 0.439219 | H |
| H9 | 1.0 | 0.633206 | 0.523465 | 0.544152 | H |
| H10 | 1.0 | 0.623490 | 0.492217 | 0.611751 | H |
| H11 | 1.0 | 0.441411 | 0.471270 | 0.573039 | H |
| H12 | 1.0 | 0.603044 | 0.327710 | 0.568296 | H |
| C1 | 1.0 | 0.576010 | 0.453975 | 0.574674 | C |
| O1 | 1.0 | 0.482589 | 0.319493 | 0.456514 | O |
| O2 | 1.0 | 0.632966 | 0.647085 | 0.454898 | O |
| O3 | 1.0 | 0.119668 | 0.590114 | 0.152744 | O |
| O4 | 1.0 | 0.117249 | 0.590767 | 0.359327 | O |
| O5 | 1.0 | 0.626910 | 0.905441 | 0.044722 | O |
| O6 | 1.0 | 0.626775 | 0.915136 | 0.258141 | O |
| O7 | 1.0 | 0.861351 | 0.415211 | 0.995009 | O |
| O8 | 1.0 | 0.872077 | 0.411700 | 0.203548 | O |
| O9 | 1.0 | 0.853343 | 0.418993 | 0.416726 | O |
| O10 | 1.0 | 0.378944 | 0.099917 | 0.094561 | O |
| O11 | 1.0 | 0.378045 | 0.082484 | 0.311465 | O |
| O12 | 1.0 | 0.918910 | 0.094138 | 0.106398 | O |
| O13 | 1.0 | 0.905683 | 0.100703 | 0.323911 | O |
| O14 | 1.0 | 0.427686 | 0.420730 | 0.995336 | O |
| O15 | 1.0 | 0.406355 | 0.415174 | 0.202513 | O |
| O16 | 1.0 | 0.404228 | 0.539084 | 0.393395 | O |
| O17 | 1.0 | 0.168888 | 0.912177 | 0.056337 | O |
| O18 | 1.0 | 0.163219 | 0.904896 | 0.262140 | O |
| O19 | 1.0 | 0.652928 | 0.574055 | 0.151643 | O |
| O20 | 1.0 | 0.672061 | 0.577329 | 0.351893 | O |
| O21 | 1.0 | 0.917910 | 0.400899 | 0.104405 | O |
| O22 | 1.0 | 0.921721 | 0.400627 | 0.317983 | O |
| O23 | 1.0 | 0.430105 | 0.083178 | 0.997887 | O |
| O24 | 1.0 | 0.405620 | 0.082237 | 0.210736 | O |
| O25 | 1.0 | 0.210782 | 0.099411 | 0.457455 | O |
| O26 | 1.0 | 0.165545 | 0.595702 | 0.054526 | O |
| O27 | 1.0 | 0.158491 | 0.595447 | 0.262470 | O |
| O28 | 1.0 | 0.652132 | 0.917404 | 0.156818 | O |
| O29 | 1.0 | 0.627177 | 0.884931 | 0.386508 | O |
| O30 | 1.0 | 0.121300 | 0.907660 | 0.154001 | O |
| O31 | 1.0 | 0.117935 | 0.914966 | 0.362639 | O |
| O32 | 1.0 | 0.625765 | 0.602105 | 0.041302 | O |
| O33 | 1.0 | 0.623248 | 0.589464 | 0.250298 | O |
| O34 | 1.0 | 0.855605 | 0.116109 | 0.995921 | O |
| O35 | 1.0 | 0.872661 | 0.087679 | 0.205558 | O |
| O36 | 1.0 | 0.873713 | 0.079283 | 0.419435 | O |
| O37 | 1.0 | 0.374520 | 0.407013 | 0.090803 | O |
| O38 | 1.0 | 0.374381 | 0.406966 | 0.299092 | O |
| O39 | 1.0 | 0.146774 | 0.250119 | 0.155443 | O |
| O40 | 1.0 | 0.178438 | 0.264193 | 0.369042 | O |

| | | | | | |
|---|---|---|---|---|---|
| O41 | 1.0 | 0.647871 | 0.257377 | 0.054727 | O |
| O42 | 1.0 | 0.657716 | 0.249536 | 0.258774 | O |
| O43 | 1.0 | 0.899771 | 0.750965 | 0.104078 | O |
| O44 | 1.0 | 0.897721 | 0.761458 | 0.315313 | O |
| O45 | 1.0 | 0.340002 | 0.753625 | 0.995223 | O |
| O46 | 1.0 | 0.372257 | 0.747469 | 0.207205 | O |
| O47 | 1.0 | 0.326936 | 0.804970 | 0.430118 | O |
| O48 | 1.0 | 0.911185 | 0.757333 | 0.994372 | O |
| O49 | 1.0 | 0.901561 | 0.748720 | 0.205271 | O |
| O50 | 1.0 | 0.913932 | 0.733431 | 0.416469 | O |
| O51 | 1.0 | 0.392965 | 0.750638 | 0.099307 | O |
| O52 | 1.0 | 0.386940 | 0.747519 | 0.309279 | O |
| O53 | 1.0 | 0.132347 | 0.250659 | 0.051806 | O |
| O54 | 1.0 | 0.120649 | 0.242758 | 0.265096 | O |
| O55 | 1.0 | 0.656409 | 0.244749 | 0.160507 | O |
| O56 | 1.0 | 0.630428 | 0.265979 | 0.368422 | O |
| O57 | 1.0 | 0.149447 | 0.453056 | 0.457008 | O |
| O58 | 1.0 | 0.558335 | 0.954826 | 0.506033 | O |
| Al1 | 1.0 | 0.404599 | 0.924666 | 0.052731 | Al |
| Al2 | 1.0 | 0.396702 | 0.926194 | 0.265061 | Al |
| Al3 | 1.0 | 0.147967 | 0.421061 | 0.108533 | Al |
| Al4 | 1.0 | 0.154435 | 0.415781 | 0.317333 | Al |
| Al5 | 1.0 | 0.647586 | 0.424493 | 0.009532 | Al |
| Al6 | 1.0 | 0.634694 | 0.421106 | 0.208904 | Al |
| Al7 | 1.0 | 0.605399 | 0.453006 | 0.420077 | Al |
| Al8 | 1.0 | 0.877685 | 0.919518 | 0.157900 | Al |
| Al9 | 1.0 | 0.873802 | 0.909642 | 0.375199 | Al |
| Al10 | 1.0 | 0.653503 | 0.078451 | 0.009998 | Al |
| Al11 | 1.0 | 0.633036 | 0.072738 | 0.211124 | Al |
| Al12 | 1.0 | 0.555080 | 0.841396 | 0.451699 | Al |
| Al13 | 1.0 | 0.877749 | 0.578853 | 0.156624 | Al |
| Al14 | 1.0 | 0.882200 | 0.572409 | 0.365846 | Al |
| Al15 | 1.0 | 0.400930 | 0.584638 | 0.049606 | Al |
| Al16 | 1.0 | 0.395624 | 0.579225 | 0.259007 | Al |
| Al17 | 1.0 | 0.151252 | 0.078551 | 0.110227 | Al |
| Al18 | 1.0 | 0.154581 | 0.091275 | 0.327359 | Al |
| Al19 | 1.0 | 0.905042 | 0.242535 | 0.050306 | Al |
| Al20 | 1.0 | 0.889378 | 0.242958 | 0.270702 | Al |
| Al21 | 1.0 | 0.515320 | 0.259253 | 0.113241 | Al |
| Al22 | 1.0 | 0.511511 | 0.250713 | 0.315791 | Al |
| Al23 | 1.0 | 0.895419 | 0.248722 | 0.159261 | Al |
| Al24 | 1.0 | 0.846512 | 0.247320 | 0.376547 | Al |
| Al25 | 1.0 | 0.112690 | 0.757479 | 0.010751 | Al |
| Al26 | 1.0 | 0.139759 | 0.751755 | 0.213823 | Al |
| Al27 | 1.0 | 0.222814 | 0.615121 | 0.419530 | Al |
| Al28 | 1.0 | 0.524952 | 0.744980 | 0.158844 | Al |
| Al29 | 1.0 | 0.514195 | 0.707386 | 0.364928 | Al |
| Al30 | 1.0 | 0.146484 | 0.750076 | 0.108068 | Al |
| Al31 | 1.0 | 0.135328 | 0.760623 | 0.315297 | Al |
| Al32 | 1.0 | 0.778756 | 0.753624 | 0.046702 | Al |
| Al33 | 1.0 | 0.758950 | 0.747739 | 0.264493 | Al |
| Al34 | 1.0 | 0.334732 | 0.252989 | 0.026774 | Al |
| Al35 | 1.0 | 0.275441 | 0.250879 | 0.216883 | Al |
| Al36 | 1.0 | 0.284089 | 0.282759 | 0.434177 | Al |

**Coordinate** for a γ-$Al_2O_3$ (100) surface with $CH_3$* and H* in Figure S8.
Absolute Energy: –750.95318799 eV.

| | |
|---|---|
| cell_length_a | 8.070750 |
| cell_length_b | 8.404430 |
| cell_length_c | 26.383160 |
| cell_angle_alpha | 90.000000 |
| cell_angle_beta | 90.000000 |
| cell_angle_gamma | 90.000000 |
| cell_volume | 1789.571263 |
| space_group_name_H-M_alt | 'P 1' |
| space_group_IT_number | 1 |

| | | | | | |
|---|---|---|---|---|---|
| H1 | 1.0 | 0.018659 | 0.441341 | 0.456484 | H |
| H2 | 1.0 | 0.396617 | 0.756631 | 0.965021 | H |
| H3 | 1.0 | 0.913047 | 0.373586 | 0.970718 | H |
| H4 | 1.0 | 0.610094 | 0.807523 | 0.487339 | H |
| H5 | 1.0 | 0.259443 | 0.851052 | 0.403042 | H |
| H6 | 1.0 | 0.172607 | 0.028528 | 0.477112 | H |
| H7 | 1.0 | 0.999976 | 0.106528 | 0.451805 | H |
| H8 | 1.0 | 0.826800 | 0.782912 | 0.444653 | H |
| H9 | 1.0 | 0.609816 | 0.494704 | 0.509246 | H |
| H10 | 1.0 | 0.668849 | 0.429763 | 0.598238 | H |
| H11 | 1.0 | 0.441206 | 0.447640 | 0.583623 | H |
| H12 | 1.0 | 0.559712 | 0.267039 | 0.562060 | H |
| C1 | 1.0 | 0.557311 | 0.382484 | 0.580671 | C |
| O1 | 1.0 | 0.439885 | 0.135680 | 0.443514 | O |
| O2 | 1.0 | 0.608765 | 0.547465 | 0.476415 | O |
| O3 | 1.0 | 0.119572 | 0.599833 | 0.155885 | O |
| O4 | 1.0 | 0.121768 | 0.592975 | 0.363410 | O |
| O5 | 1.0 | 0.627135 | 0.911220 | 0.041974 | O |
| O6 | 1.0 | 0.627002 | 0.923190 | 0.258495 | O |
| O7 | 1.0 | 0.874551 | 0.420502 | 0.002429 | O |
| O8 | 1.0 | 0.870043 | 0.416423 | 0.206732 | O |
| O9 | 1.0 | 0.858386 | 0.431637 | 0.415948 | O |
| O10 | 1.0 | 0.379939 | 0.103152 | 0.095185 | O |
| O11 | 1.0 | 0.376632 | 0.093406 | 0.312637 | O |
| O12 | 1.0 | 0.917596 | 0.101707 | 0.108880 | O |
| O13 | 1.0 | 0.908708 | 0.106270 | 0.320023 | O |
| O14 | 1.0 | 0.406429 | 0.427847 | 0.999530 | O |
| O15 | 1.0 | 0.402956 | 0.427771 | 0.210723 | O |
| O16 | 1.0 | 0.393644 | 0.430909 | 0.411054 | O |
| O17 | 1.0 | 0.167517 | 0.916115 | 0.058191 | O |
| O18 | 1.0 | 0.160761 | 0.914427 | 0.265007 | O |
| O19 | 1.0 | 0.651847 | 0.586313 | 0.157272 | O |
| O20 | 1.0 | 0.644632 | 0.606122 | 0.370795 | O |
| O21 | 1.0 | 0.917723 | 0.410806 | 0.108311 | O |
| O22 | 1.0 | 0.912397 | 0.415169 | 0.317811 | O |
| O23 | 1.0 | 0.422548 | 0.083041 | 0.997688 | O |
| O24 | 1.0 | 0.403253 | 0.089844 | 0.212260 | O |
| O25 | 1.0 | 0.118965 | 0.128333 | 0.468248 | O |
| O26 | 1.0 | 0.165103 | 0.600909 | 0.058219 | O |
| O27 | 1.0 | 0.159273 | 0.605512 | 0.265935 | O |
| O28 | 1.0 | 0.650616 | 0.928006 | 0.157993 | O |
| O29 | 1.0 | 0.644553 | 0.933446 | 0.372563 | O |
| O30 | 1.0 | 0.120761 | 0.914754 | 0.155706 | O |
| O31 | 1.0 | 0.121626 | 0.925044 | 0.363199 | O |
| O32 | 1.0 | 0.625053 | 0.598836 | 0.039273 | O |
| O33 | 1.0 | 0.626933 | 0.593122 | 0.257710 | O |
| O34 | 1.0 | 0.866154 | 0.110763 | 0.999445 | O |
| O35 | 1.0 | 0.869907 | 0.098680 | 0.207084 | O |
| O36 | 1.0 | 0.856272 | 0.096967 | 0.419399 | O |
| O37 | 1.0 | 0.378054 | 0.418458 | 0.098018 | O |
| O38 | 1.0 | 0.372528 | 0.421724 | 0.308622 | O |
| O39 | 1.0 | 0.146801 | 0.256840 | 0.157136 | O |
| O40 | 1.0 | 0.138420 | 0.258409 | 0.369361 | O |

| | | | | | |
|---|---|---|---|---|---|
| O41 | 1.0 | 0.650324 | 0.261917 | 0.054156 | O |
| O42 | 1.0 | 0.652586 | 0.257939 | 0.260915 | O |
| O43 | 1.0 | 0.894606 | 0.756826 | 0.105122 | O |
| O44 | 1.0 | 0.894002 | 0.759586 | 0.317537 | O |
| O45 | 1.0 | 0.337574 | 0.757889 | 0.997131 | O |
| O46 | 1.0 | 0.374127 | 0.757712 | 0.210583 | O |
| O47 | 1.0 | 0.345146 | 0.770613 | 0.416247 | O |
| O48 | 1.0 | 0.913754 | 0.756619 | 0.994484 | O |
| O49 | 1.0 | 0.902060 | 0.757962 | 0.206904 | O |
| O50 | 1.0 | 0.906001 | 0.763497 | 0.415806 | O |
| O51 | 1.0 | 0.395535 | 0.757413 | 0.100705 | O |
| O52 | 1.0 | 0.387564 | 0.760003 | 0.312053 | O |
| O53 | 1.0 | 0.131864 | 0.255772 | 0.055817 | O |
| O54 | 1.0 | 0.122257 | 0.256447 | 0.265624 | O |
| O55 | 1.0 | 0.648912 | 0.256938 | 0.161991 | O |
| O56 | 1.0 | 0.645380 | 0.275047 | 0.369947 | O |
| O57 | 1.0 | 0.141894 | 0.446816 | 0.462597 | O |
| O58 | 1.0 | 0.680925 | 0.897137 | 0.478258 | O |
| Al1 | 1.0 | 0.406235 | 0.928963 | 0.051925 | Al |
| Al2 | 1.0 | 0.398017 | 0.934822 | 0.264729 | Al |
| Al3 | 1.0 | 0.150562 | 0.428572 | 0.109922 | Al |
| Al4 | 1.0 | 0.146215 | 0.423928 | 0.317330 | Al |
| Al5 | 1.0 | 0.643734 | 0.422636 | 0.001164 | Al |
| Al6 | 1.0 | 0.631146 | 0.432472 | 0.210985 | Al |
| Al7 | 1.0 | 0.618220 | 0.446698 | 0.417087 | Al |
| Al8 | 1.0 | 0.876516 | 0.928446 | 0.157049 | Al |
| Al9 | 1.0 | 0.881298 | 0.932924 | 0.370419 | Al |
| Al10 | 1.0 | 0.655377 | 0.088956 | 0.006114 | Al |
| Al11 | 1.0 | 0.631458 | 0.082842 | 0.211296 | Al |
| Al12 | 1.0 | 0.629114 | 0.042928 | 0.432439 | Al |
| Al13 | 1.0 | 0.877531 | 0.585657 | 0.156945 | Al |
| Al14 | 1.0 | 0.873272 | 0.595735 | 0.370579 | Al |
| Al15 | 1.0 | 0.403281 | 0.582318 | 0.051935 | Al |
| Al16 | 1.0 | 0.398286 | 0.584928 | 0.263727 | Al |
| Al17 | 1.0 | 0.151614 | 0.086111 | 0.110169 | Al |
| Al18 | 1.0 | 0.149257 | 0.092855 | 0.320634 | Al |
| Al19 | 1.0 | 0.901074 | 0.249687 | 0.050123 | Al |
| Al20 | 1.0 | 0.885393 | 0.257991 | 0.267627 | Al |
| Al21 | 1.0 | 0.520553 | 0.262018 | 0.110102 | Al |
| Al22 | 1.0 | 0.512509 | 0.260087 | 0.318156 | Al |
| Al23 | 1.0 | 0.892419 | 0.257243 | 0.159697 | Al |
| Al24 | 1.0 | 0.877104 | 0.263165 | 0.373740 | Al |
| Al25 | 1.0 | 0.112918 | 0.759456 | 0.012585 | Al |
| Al26 | 1.0 | 0.140009 | 0.759069 | 0.214470 | Al |
| Al27 | 1.0 | 0.245611 | 0.577827 | 0.420652 | Al |
| Al28 | 1.0 | 0.521713 | 0.756699 | 0.159419 | Al |
| Al29 | 1.0 | 0.514481 | 0.769730 | 0.368375 | Al |
| Al30 | 1.0 | 0.147397 | 0.756941 | 0.109414 | Al |
| Al31 | 1.0 | 0.138547 | 0.762609 | 0.317648 | Al |
| Al32 | 1.0 | 0.775033 | 0.754565 | 0.045179 | Al |
| Al33 | 1.0 | 0.761861 | 0.757790 | 0.263305 | Al |
| Al34 | 1.0 | 0.323506 | 0.250956 | 0.023721 | Al |
| Al35 | 1.0 | 0.273407 | 0.258909 | 0.215559 | Al |
| Al36 | 1.0 | 0.268238 | 0.260468 | 0.431301 | Al |
